# Molecular insights into disease-associated glutamate transporter (EAAT1 / *SLC1A3*) variants using *in silico* and *in vitro* approaches

**DOI:** 10.3389/fmolb.2023.1286673

**Published:** 2023-11-23

**Authors:** Marina Gorostiola González, Hubert J. Sijben, Laura Dall’ Acqua, Rongfang Liu, Adriaan P. IJzerman, Laura H. Heitman, Gerard J. P. van Westen

**Affiliations:** ^1^ Division of Drug Discovery and Safety, Leiden Academic Centre for Drug Research, Leiden University, Leiden, Netherlands; ^2^ Oncode Institute, Leiden, Netherlands

**Keywords:** EAAT1, SLC1A3, glutamate transporter, variants, phenotypic assay, molecular dynamics, docking

## Abstract

Glutamate is an essential excitatory neurotransmitter and an intermediate for energy metabolism. Depending on the tumor site, cancer cells have increased or decreased expression of excitatory amino acid transporter 1 or 2 (EAAT1/2, *SLC1A3/2*) to regulate glutamate uptake for the benefit of tumor growth. Thus, EAAT1/2 may be an attractive target for therapeutic intervention in oncology. Genetic variation of EAAT1 has been associated with rare cases of episodic ataxia, but the occurrence and functional contribution of EAAT1 mutants in other diseases, such as cancer, is poorly understood. Here, 105 unique somatic EAAT1 mutations were identified in cancer patients from the Genomic Data Commons dataset. Using EAAT1 crystal structures and *in silico* studies, eight mutations were selected based on their close proximity to the orthosteric or allosteric ligand binding sites and the predicted change in ligand binding affinity. *In vitro* functional assessment in a live-cell, impedance-based phenotypic assay demonstrated that these mutants differentially affect L-glutamate and L-aspartate transport, as well as the inhibitory potency of an orthosteric (TFB-TBOA) and allosteric (UCPH-101) inhibitor. Moreover, two episodic ataxia-related mutants displayed functional responses that were in line with literature, which confirmed the validity of our assay. Of note, ataxia-related mutant M128R displayed inhibitor-induced functional responses never described before. Finally, molecular dynamics (MD) simulations were performed to gain mechanistic insights into the observed functional effects. Taken together, the results in this work demonstrate 1) the suitability of the label-free phenotypic method to assess functional variation of EAAT1 mutants and 2) the opportunity and challenges of using *in silico* techniques to rationalize the *in vitro* phenotype of disease-relevant mutants.

## 1 Introduction

Glutamate is an abundant endogenous amino acid that acts as the major excitatory neurotransmitter in the central nervous system and serves as a key metabolite in energy homeostasis (Tzingounis and Wadiche, 2007). In the synaptic cleft glutamate is transported across the cell membrane via excitatory amino acid transporters (EAATs), which belong to subfamily 1 of the solute carrier (SLC) transporters (Vandenberg and Ryan, 2013). Glutamate transport is thermodynamically coupled to the transport of three Na^+^ ions and one proton, and the counter-transport of one K^+^ ion, where binding of Na^+^ and/or substrate activates an uncoupled Cl^−^ conductive state (Alleva et al., 2022). Deregulated glutamate levels have been associated with a plethora of neurological diseases (Lewerenz and Maher, 2015; Peterson and Binder, 2020) and more recently with cancer (Freidman et al., 2020; Yi et al., 2020). As a result, pharmacological modulation of EAATs may be a promising therapeutic strategy for conditions that are associated with altered glutamate levels (A. A. Jensen et al., 2009; Kortagere et al., 2018).

Depending on the location of the tumor, cancerous cells have been shown to exploit the uptake, metabolism and signaling properties of glutamate as well as aspartate as fuel for tumor proliferation and expansion. Healthy glia cells abundantly express EAAT1 and EAAT2 to mediate the majority of glutamate clearance (Vandenberg and Ryan, 2013). However, expression levels of EAAT2 are vastly reduced in gliomas, which combined with increased efflux via the glutamate/cystine antiporter (xCT, *SLC7A11*) leads to elevated glutamate levels surrounding the glioma that induce cell death and allow further growth of the tumor (Takano et al., 2001; Robert and Sontheimer, 2014). Moreover, EAAT1 was found to be overexpressed and cause glutamate efflux in aggressive glioblastomas, which indicates selective EAAT1 inhibitors as a potential treatment option for glioma (Corbetta et al., 2019). In several instances of cancer in peripheral tissues EAAT1 expression has been linked to a poor disease prognosis. Under hypoxia or conditions that starve the tumor of glutamine, some cancer cells promote EAAT1 or EAAT2 expression to drive uptake of aspartate or glutamate which rescues cancer cell growth (Garcia-Bermudez et al., 2018; Tajan et al., 2018; Bacci et al., 2019). As such, EAAT expression in such tumors could be a predictive biomarker and pharmacological modulation of glutamate transporter expression or activity could be of therapeutic interest.

Despite the clear advantages for tumor cells to regulate EAAT expression, little is known about human genetic variations of these transporters in cancer, although several mutations have been associated with other diseases. Thus far, reports have linked seven missense mutations in the coding region of EAAT1 to the etiology of extremely rare cases of episodic ataxia type 6 (EA6) (Chivukula et al., 2020). These mutants vary in their degree of loss- or gain-of-function of substrate transport and/or anion conductivity (Chivukula et al., 2020). Moreover, several other EAAT1 mutations and duplications have been associated with other neurological disorders including migraine, ADHD, autism, and Tourette’s syndrome (Adamczyk et al., 2011; van Amen-Hellebrekers et al., 2016; Kovermann et al., 2017). To the best of our knowledge, there have been no reports so far that associate mutations of EAAT1 to the development and progression of cancer.

Over the last 15 years, a growing number of 3D structures have been published for the archaeal glutamate transporter orthologues Glt_Ph_ (Boudker et al., 2007) and Glt_Tk_ (Guskov et al., 2016), as well as human EAAT1 (Canul-Tec et al., 2017; Canul-Tec et al., 2022), EAAT2 (Kato et al., 2022) and EAAT3 (Qiu et al., 2021), in complex with the endogenous substrate L-aspartate, Na^+^ ions and/or inhibitors. Glutamate transporters assemble in obligate homo-trimers of which the protomers operate independently of each other. Each protomer consists of a rigid trimerization or scaffold domain (scaD) and a dynamic transport domain (tranD) that engages with the substrate and co-transported Na^+^ ions (Canul-Tec et al., 2017). Structures covering inward-facing, intermediate, and outward-facing conformations provide information on the movement of individual transmembrane helices (TMs). Specifically, the flexible helical hairpin 2 (HP2) in tranD controls the access of ligands to the substrate binding site and is an essential ‘gate’ that upon opening and closing regulates the ‘elevator-like’ translocation of tranD. Of note, these transport mechanisms have been elucidated in part thanks to molecular dynamic (MD) simulations (Kortzak et al., 2019; Alleva et al., 2020). Thus, these structures may be used to gain mechanistic insight into the effects of genetic variability on transport function, as was previously demonstrated by mapping genetic variants of glucose (GLUT1) and nucleoside (ENT1) transporters to their respective crystal structures (Schaller and Lauschke, 2019).

In this study, a series of EAAT1 somatic mutations that were identified from biopsy material of cancer patients represented in the Genomic Data Commons (GDC) dataset (M. A. Jensen et al., 2017) were characterized. Using the reported ligand-bound crystal structures of EAAT1 (Canul-Tec et al., 2017; Canul-Tec et al., 2022), predictions were made on which variants would most likely impact binding of substrates (L-glutamate and L-aspartate). To determine whether these mutants would affect the binding of potential pharmacological modulators, the orthosteric inhibitor TFB-TBOA (Shimamoto et al., 2004) and the allosteric inhibitor UCPH-101 (A. A. Jensen et al., 2009) were included, which have been co-crystalized with EAAT1 (Canul-Tec et al., 2017). The selected eight mutations, together with two EA6-associated mutants (M128R, T318A), were tested *in vitro* for substrate uptake and inhibition using a label-free impedance-based phenotypic assay that was previously developed in our lab (Sijben et al., 2022). Mutants displayed divergent effects on EAAT1 function, which was apparent from an altered substrate and/or inhibitor potency. Finally, MD simulations and molecular docking were used to explore the mechanisms of the observed *in vitro* results. These *in silico* approaches mainly explored the effect of conformational changes on ligand and ion coordination stability. We demonstrate the application of a combined *in silico* and *in vitro* approach to characterize EAAT1 variants, which could aid drug discovery efforts.

## 2 Materials and methods

### 2.1 Materials

Modified Jump In T-REx HEK 293 (JumpIn) cells overexpressing human wild-type (WT, EAAT1_WT_) or mutant EAAT1 (see section 2.6–2.9) were kindly provided by the RESOLUTE consortium (Research Center for Molecular Medicine, Medical University of Vienna, Austria). L-glutamic acid monosodium salt monohydrate, L-aspartic acid monosodium salt monohydrate, doxycycline hyclate, Dulbecco’s modified Eagle’s medium (DMEM) and Dulbecco’s phosphate-buffered saline (PBS) were purchased from Sigma Aldrich (St. Louis, MO, United States). 2-amino-4-(4-methoxyphenyl)-7-(naphthalen-1-yl)-5-oxo-5,6,7,8-tetrahydro-4H-chromene-3-carbonitrile (UCPH-101) was purchased from Santa Cruz Biotechnology (Dallas, TX, United States). (2S,3S)-3-[3-[4-(trifluoromethyl)benzoylamino]benzyloxy] aspartate (TFB-TBOA) was purchased from Axon Medchem (Groningen, Netherlands). Lipofectamine 3,000, P3000 buffer, Gateway LR Clonase II enzyme mix and Proteinase K solution were purchased from ThermoFischer (Waltham, MA, United States). QuikChange II kit was purchased from Agilent Technologies (Santa Clara, CA, United States). QIAprep Spin Miniprep Kit was purchased from QIAGEN (Hilden, Germany). xCELLigence PET E-plates 96 (Agilent Technologies, Santa Clara, CA, United States) were purchased from Bioké (Leiden, Netherlands). All other chemicals were of analytical grade and obtained from standard commercial sources.

### 2.2 Selection of cancer-related mutations

Cancer-related mutations were obtained from the Genomic Data Commons (M. A. Jensen et al., 2017) version 22.0 released on 16 January 2020, as re-compiled by Bongers et al. (2022). Somatic missense mutations were retrieved for gene *SLC1A3* (EAAT1) in all cancer types. The 105 unique mutations found were mapped onto the 3D structure of EAAT1 [PDB 5LLU, 5MJU (Canul-Tec et al., 2017) and 7AWM (Canul-Tec et al., 2022)], with particular attention to the functional motifs and binding sites defined by Canul-Tec et al. (2017); Canul-Tec et al. (2022) Two sets of mutations of interest were defined by visual inspection in the proximity (i.e., 5 Å from co-crystalized ligands) of the orthosteric binding site—occupied by the substrate L-aspartate—and allosteric binding site—occupied by allosteric inhibitor UCPH-101. The ‘orthosteric’ set of mutations included P392L, A446E, A446V, L448Q, and R479W. The ‘allosteric’ set of mutations included Y127C, C252F, R388K, F389L, V390M, and I397V. Additionally, mutation V247F is located at the interface of the two sites and was therefore included in both sets.

As reference, *SLC1A3* (EAAT1) mutations found in natural variance in the 1,000 Genomes dataset (Auton et al., 2015) were retrieved. This dataset was obtained from the Uniprot variance database in October 2020 (The UniProt Consortium, 2019). For the purpose of comparison, the percentage of mutations in EAAT1 found in cancer patients and natural variance was calculated by dividing the number of mutations in EAAT1 by the number of patients in each dataset (10,179 and 3,202, respectively) and multiplying it by 100%.

### 2.3 System preparation and molecular docking

The monomeric EAAT1 systems for binding affinity change predictions were prepared from chain A in PDB codes 5LLU and 5MJU (Canul-Tec et al., 2017) in ICM-Pro version 3.9-2c (Molsoft LLC, San Diego) (Abagyan et al., 1994; Neves et al., 2012). The systems were prepared by optimizing the protonation states and orientation of histidine and cysteine residues, and the orientation of glutamine and asparagine residues. Moreover, the position of hydrogen atoms was sampled and optimized. Stabilizing mutations in residues selected for further analysis were reverted (i.e., C252V, T318M). Subsequently, L-glutamate was prepared by adding hydrogen atoms and assigning atomic charges and docked it into the orthosteric binding site of PDB 5LLU, originally occupied by L-aspartate. Upon removal of L-aspartate from the binding site, docking was performed with default settings and 10 poses stored by defining the residues surrounding L-aspartate as the binding site. The poses were analyzed in light of the experimental data available, docking scores, and interaction patterns. The pose with the highest docking score was selected for further analysis.

EAAT1 trimeric systems with L-Aspartate bound were prepared for MD simulations from the biological assembly of PDB 7AWM, containing chains A-C. This preparation step was performed directly in academic version of the Desmond program, release 2021.1 (Bowers et al., 2006), and is described in detail in the corresponding MD section.

### 2.4 Binding affinity change predictions

To prioritize mutations for *in vitro* testing, changes in EAAT1 binding affinity were predicted to endogenous substrates L-aspartate and L-glutamate, and the inhibitors TFB-TBOA (competitive) and UCPH-101 (allosteric) caused by point mutations. This analysis was performed in ICM-Pro as follows. The difference in binding energy (ΔΔG_bind_, in kcal/mol) is calculated as the difference between the Gibbs binding energy (ΔG_bind_, in kcal/mol) in the mutant and the WT. ΔG_bind_ is calculated for fixed backbone and Monte Carlo-sampled flexible side chains in the vicinity of the mutated residue as the energy of the protein-ligand complex minus the energy of the protein and ligand separately.

For the cancer-related mutations found in the orthosteric binding site (P392L, A446E, A446V, L448Q, and R479W), ΔΔG_bind_ was calculated for endogenous ligands L-aspartate and L-glutamate (previously docked) in system 5LLU. Moreover, ΔΔG_bind_ was calculated for the competitive inhibitor TFB-TBOA in system 5MJU. For the cancer-related mutations found in the allosteric binding site (Y127C, C252F, R388K, F389L, V390M, and I397V), ΔΔG_bind_ was calculated for the allosteric inhibitor UCPH-101 in system 5MJU. For V247F, which is at the interface of both ligand binding sites, ΔΔG_bind_ was calculated for L-glutamate, L-aspartate, TFB-TBOA and UCPH-101 as described above.

### 2.5 Structural visualization

All visualizations of EAAT1 structures were generated in PyMOL using PDB 7AWM. Where TFB-TBOA was visualized, PDB 5MJU was superimposed on 7AWM.

### 2.6 Mutagenesis

DNA primers for EAAT1 mutants were designed with a single or double base pair substitution for the resultant amino acid using the QuikChange Primer Design Program and synthesized by Integrated DNA Technologies (IDT, Leuven, Belgium) (Table 1). Site-directed mutagenesis was performed using a QuikChange II kit. In brief, per mutant 50 ng template DNA (codon-optimized ORF for EAAT1 (*SLC1A3*) in a pDONR221 vector (pDONR221-*SLC1A3*, Addgene #131889)) together with 10 µM forward and reverse primer, 1 µL dNTP mix, 2.5 µL 10x reaction buffer and 2.5 U DNA polymerase were run in a PCR thermal cycler for 22 cycles (each cycle consisted of 30 s 95°C, 1 min 55°C, 10 min 68°C). Non-mutated DNA was removed by addition of 5 U DpnI restriction enzyme for 2 h at 37°C. Mutant DNA was transformed into XL1-Blue competent cells in the presence of 50 μg/mL kanamycin for selection. Plasmid was isolated using a QIAprep Spin Miniprep Kit verified by Sanger sequencing (Leiden Genome Technology Center, Leiden, Netherlands).

**TABLE 1:** DNA primers (forward and reverse) that were used to generate eight cancer-related and two ataxia-related EAAT1 mutants. Mutated bases are bold and underlined.

Mutant	Forward primer (5’)	Reverse primer (5’)
Y127C	GAGAGCCGTGGTGTACT **G** TATGACCACAACCATCA	TGATGGTTGTGGTCATA **C** AGTACACCACGGCTCTC
M128R	TGAGAGCCGTGGTGTACTATA **G** GACCACAACCAT	ATGGTTGTGGTC **C** TATAGTACACCACGGCTCTCA
V247F	AATGCCCTGGGCCTG **T** T **C** GTGTTCAGCATGTGC	GCACATGCTGAACAC **G** A **A** CAGGCCCAGGGCATT
T318A	CAGCTGGCCATGTAC **G** CCGTGACAGTGATCG	CGATCACTGTCACGG **C** GTACATGGCCAGCTG
V390M	GACAAGCGGGTGACCAGATTT **A** TGCTGCCAGTG	CACTGGCAGCA **T** AAATCTGGTCACCCGCTTGTC
P392L	CAGATTTGTGCTGC **T** AGTGGGCGCCACCA	TGGTGGCGCCCACT **A** GCAGCACAAATCTG
A446E	CAGGCATCCCACAGG **AA** GGCCTGGTGACCATG	CATGGTCACCAGGCC **TT** CCTGTGGGATGCCTG
A446V	GCATCCCACAGG **T** CGGCCTGGTGAC	GTCACCAGGCCG **A** CCTGTGGGATGC
L448Q	CACAGGCCGGCC **A** GGTGACCATGGT	ACCATGGTCACC **T** GGCCGGCCTGTG
R479W	GGTTTCTGGATAGGCTG **T** G **G** ACAACCACAAACGTGCT	AGCACGTTTGTGGTTGT **C** C **A** CAGCCTATCCAGAAACC

### 2.7 Gateway cloning

To allow stable transfection into JumpIn cells, the WT and mutant pDONR221-*SLC1A3* plasmids were cloned into a pJTI R4 DEST CMV TO pA expression vector with a C-terminal Twin-Strep-tag and a hemagglutinin (HA)-tag using Gateway cloning. The expression vector contains a tet-operon (TO) that allows doxycycline (dox)-inducible expression of the transgene. In brief, 150 ng pDONR221-*SLC1A3* plasmid and 150 ng pJTI R4 DEST CMV TO pA in TE buffer (10 mM Tris, 1 mM EDTA) were incubated with Gateway LR Clonase II enzyme mix at 25°C for 1 h. To remove endogenous nucleases, the mixture was incubated with a Proteinase K solution for 10 min at 37°C. The resulting vectors (WT or mutant pJTI-*SLC1A3*) were transformed into XL1-Blue competent cells in the presence of 100 μg/mL ampicillin for selection. Plasmid was isolated and sequenced as described in the previous section.

### 2.8 Cell culture

JumpIn-EAAT1 cells were split twice per week to 10 cm dishes in culture medium (high glucose DMEM containing 10% fetal calf serum, 2 mM Glutamax, 100 IU/mL penicillin and 100 μg/mL streptomycin) at 37°C and 5% CO_2_. After thawing and recovery, cells were grown for 3–5 days in culture medium with 5 μg/mL blasticidin and 2 mg/mL G418 before switching to culture medium.

### 2.9 Generation of stably transfected WT and mutant JumpIn-EAAT1 cells

JumpIn cells were seeded at 90,000 cells/well in culture medium onto a 24-well culture plate and grown within 24 h to 60%–70% confluence. Per mutant or WT, a mix of 1.8 µL P3000 buffer, 450 µg pJTI R4 Integrase plasmid and 450 µg pJTI-*SLC1A3* plasmid in OptiMEM was added to a mix of 2.1 µL Lipofectamine 3,000 in OptiMEM (90 µL total per condition) and incubated for 5 min at RT. As a control for antibiotic selection, one dish of cells was incubated with sterile water instead of pJTI-*SLC1A3*. Cells were transfected with 60 µL of the total mix. On the next day the transfection medium was replaced by fresh culture medium. After 24 h cells were trypsinized and seeded onto 6 cm culture dishes at 200,000 cells/well to grow for 3–4 days. When 70% confluence was reached medium was replaced with selection medium (culture medium with 1 mg/mL G418) to select for successfully transfected cells. Selection medium was refreshed every 2–3 days for 2 weeks until non-transfected cells were all dead and colonies had grown in the transfected dishes. Colonies were resuspended in selection medium and grown to confluence before cryofreezing pools of transfected cells. Prior to use in experiments, cells were cultured in regular culture medium for at least 24 h.

### 2.10 Whole cell HA-tag ELISA

To determine the relative amount of C-terminal HA-tagged protein expressed in doxycycline (dox)-induced JumpIn-EAAT1 WT and mutant cells, an enzyme-linked immunosorbent assay (ELISA) was performed on whole, permeabilized cells. Each condition was tested in quintuplicate per experiment. Cells were seeded in culture medium onto a 96-well culture plate coated with 0.1 mg/mL poly-D-lysine at 60,000 cells/well in the presence or absence of 1 μg/mL dox (100 µL total volume) and were grown for 22–24 h at 37°C and 5% CO_2_. Cells were washed with PBS and fixed with 4% formaldehyde for 10 min, then washed with Tris-buffered saline (TBS). To allow access of the antibodies to the intracellular HA-tag, cells were incubated with permeabilization buffer (TBS +0.5% Tween-20 (TBST), 2% bovine serum albumin (BSA) and 0.2% saponin) for 60 min at RT. After blocking and permeabilization, cells were incubated with 1:2,500 rabbit anti-HA polyclonal antibody (Invitrogen, Carlsbad, CA, United States) for 60 min at RT and washed with TBST. Subsequently, cells were incubated for with 1:3,000 goat anti-rabbit horse radish peroxidase (HRP)-conjugated IgG antibody (Brunschwig Chemie, Amsterdam, Netherlands) for 30 min at RT and washed with TBS. Immunoreactivity was visualized by addition of 3,3′,5,5′-tetramethylbenzidine (TMB) for 2.5 min at RT and subsequent quenching with 1 M H_3_PO_4_. Absorbance was measured at 450 nm using a Wallac EnVision multimode plate reader (PerkinElmer, Groningen, Netherlands).

### 2.11 Impedance-based phenotypic assay

To measure functional substrate responses and substrate inhibition on WT and mutant JumpIn-EAAT1 cells, a label-free impedance-based cell swelling assay was employed as described previously by our lab (Sijben et al., 2022). An xCELLigence real-time cell analyzer (RTCA) system (Agilent Technologies, Santa Clara, CA, United States) was used to record real-time changes in cell morphology. The assay principle is that EAAT1-mediated, Na^+^-dependent substrate influx induces cell swelling, which leads to cell spreading. This results in an increased cellular impedance over time and as such is a readout of transporter function. For the assay, JumpIn-EAAT1 cells are cultured in medium onto gold-plated electrodes of a 96-well E-plate and for each well the impedance is measured on predefined time intervals at 10 kHz. The impedance is converted to the unitless parameter Cell Index (CI), which can be plotted over time:

$$CI=\frac{\left(Z_{i}-Z_{0}\right)\Omega }{15\Omega }$$

where Z_i_ is the impedance at any given time point and Z_0_ is the baseline impedance measured at the start of each experiment (Kho et al., 2015).

Assays were performed at 37°C and 5% CO_2_ in a final volume of 100 µL/well. Baseline impedance was measured in 40 µL culture medium prior to cell seeding. Cells grown to 70%–80% confluence were seeded in 50 μL at 60,000 cells/well in the presence of 1 μg/mL dox to induce EAAT1 expression and left at RT for 30 min prior to placement of the E-plate in the RTCA recording station. After 22 h, cells were pretreated with 5 µL vehicle (PBS/DMSO) or, in inhibitor experiments, 1 nM–10 µM of TFB-TBOA or UCPH-101 or 1 µM ouabain, and impedance was recorded for 60 min. Subsequently, cells were stimulated with 5 µL vehicle (PBS), 10 μM–1 mM L-glutamate (submaximal concentration [EC_80_, 1 mM] in inhibitor experiments) or L-aspartate, 200 nM TFB-TBOA (EC_50_) or 6.3 µM UCPH-101 (EC_50_), and impedance was recorded for 120 min. Each condition was tested in duplicate per experiment and levels of DMSO were kept constant at 0.1% for all assays and wells.

### 2.12 Data analysis and statistics

#### 2.12.1 Whole cell HA-tag ELISA

In each experiment, the mean absorbance for each condition was divided over the mean absorbance of non-induced (–dox) JumpIn-EAAT1_WT_ cells to obtain fold expression over–dox cells. To assess whether total protein expression of dox-induced (+dox) JumpIn-EAAT1 mutant cells was significantly different from +dox JumpIn-EAAT1_WT_ cells, a one-way ANOVA with Dunnett’s *post hoc* test was done for cells that were tested on the same ELISA plate.

#### 2.12.2 Impedance-based phenotypic assay

Data was recorded using RTCA Software v2.0 or v2.1.1 (ACEA Biosciences). Depending on the part that was used for analysis, the CI values were normalized to the time of inhibitor pretreatment or substrate stimulation yielding normalized CI (nCI) values for all subsequent data points. The nCI values were exported and analyzed in GraphPad Prism v9 (GraphPad Software, San Diego, CA, United States). Vehicle-only conditions were subtracted from all other conditions to correct for vehicle-induced, ligand-independent effects. The remaining nCI curves were quantified by analyzing the net area under the curve (AUC) of the first 120 min after substrate stimulation. The AUC values, which are expressed as the cellular response, were fitted to a sigmoidal concentration-effect curve with a variable slope to determine the potencies of the EAAT1 substrates and inhibitors. Data are shown as the mean ± standard error of the mean (SEM) of at least three separate experiments each performed in duplicate, unless stated otherwise. Comparison of multiple mean values to a control (i.e., EAAT1_WT_) was done using a one-way ANOVA with Dunnett’s *post hoc* test. Differences were considered statistically significant when *p*-values were below 0.05.

### 2.13 Molecular dynamics

Conformational changes were sampled over time in WT and mutant EAAT1 trimeric systems with MD simulations. The simulations were performed using the academic version of the Desmond program, release 2021.1 (Bowers et al., 2006). The OPLS-2005 force field and SPC water model were used. EAAT1 was simulated with L-Aspartate bound as substrate, directly derived from PDB 7AWM biological assembly, where the co-crystalized substrate L-Aspartate and Na^+^ ions were kept during preparation and UCPH-101 and Ba^2+^ ions were removed. WT and seven mutants with differential *in vitro* results (Y127C, M128R, P392L, A446E, A446V, L448Q, and R479W) were sampled. All systems were prepared in four steps: (a) the mutation of interest was introduced; (b) default protein preparation wizard was run; (c) the system was stripped to contain the protein trimer and the ligands and ions of interest as defined for each type of system i-iii; (d) the system was embedded in a POPC lipid bilayer respect to the α-helices, solvated with SPC water molecules, the charge was neutralized with Cl^−^ ions, and NaCl was added in physiological concentration (0.15 M). Subsequently, the systems were relaxed with the default protocol, which includes a restrained minimization followed by an unrestrained minimization and four stages of MD runs with decreasing constraints. The production runs were simulated for 500 ns with a recording interval of 500 ps (1,000 frames) in an NPT ensemble with temperature 300 K and pressure 1 bar. Each system was run for ten replicates with velocities randomly initialized with random seeds (Supplementary Table S1).

### 2.14 MD trajectory analysis

The analysis of MD trajectories was performed in Desmond and PyMOL version 2.5.2 (Schrödinger LTD.). Using Desmond analysis scripts, the trajectories’ Root Mean Square Deviation (RMSD) was calculated for the protein α carbon (Cα) atoms and for the ligand (L-Aspartate) with respect to the protein. These RMSD values represent the stability of the protein system and the ligand, respectively, over the time of the simulation. Moreover, Root Mean Square Fluctuation (RMSF) was calculated for the protein Cα atoms. The RMSF values represent the stability/flexibility through the simulation of each of the protein residues. RMSD and RMSF values were calculated independently for each chain in the trimeric system. Protein RMSD was used as an overall measure of the system’s stability. Therefore (chain) systems with protein RMSD reaching 10 Å were excluded from further analysis.

In PyMOL, the trajectories were loaded and fitted to the first frame in the simulation to correct for rotations and translations. Subsequently, the distance in each frame was calculated between a pair of atoms to obtain four measures (1–4). (1) HP2 opening: distance between HP1 and HP2 domain tips, as defined by Alleva *et al.* for Glt_Ph_ (Alleva et al., 2020). In EAAT1, the distance was measured between S366 Cα (HP1 tip) and G442 Cα (HP2 tip). The atoms corresponding to the HP1 and HP2 domain tips in EAAT1 were defined via sequence alignment with Glt_Ph_. (2) Na^+^ coordination in Na1 site: distance between Na^+^ ion originally coordinated in Na1 site and one of the Na1 coordinating atoms. The distance was measured between Na^+^ with residue number 601 and D487 Cα. (3) Na^+^ coordination in Na2 site: distance between Na^+^ ion originally coordinated in Na2 site and one of the Na2 coordinating atoms. The distance was measured between Na^+^ with residue number 603 and T396 Cα. (4) Na^+^ coordination in Na3 site: distance between Na^+^ ion originally coordinated in Na3 site and one of the Na3 coordinating atoms. The distance was measured between Na^+^ with residue number 602 and D400 Cα. These distances were measured independently for each chain in the trimeric system.

Sampling density of MD metrics computed per frame (i.e., RMSD and distances) was plotted in Python 3.8 using Matplotlib and Seaborn libraries (Hunter, 2007; Van Rossum and Drake, 2009; Waskom, 2021). The sampling density maps were calculated with data from the 1,002 frames in each chain (A,B,C) sampled in the ten replicates simulated per mutant. Unstable systems (i.e., protein RMSD reaching 10 Å) were not included in the density maps. These included Y127C replicate 9 (all chains) and replicate 10 (chain A); M128R replicate 1 (all chains); P392L replicate 1 (chain C) and replicate 10 (chain C); A446E replicate 3 (chain B) and replicate 4 (all chains); A446V replicate 3 (all chains); L448Q replicate 4 (chain C); and R479W replicate 3 (chain A), replicate 5 (chain A), and replicate 7 (replicates A, B).

### 2.15 Inhibitor docking in MD trajectory frames

The orthosteric (TFB-TBOA) and allosteric (UCPH-101) inhibitors were docked in a representative selection of MD frames with the most frequent HP2 opening distances but different binding pocket conformations. Chain A was selected for docking because it showed the highest substrate stability in EAAT1_WT_. For EAAT1_WT_ and each simulated mutant, five random frames with the most frequent HP2 opening distances in the distribution across all replicates and frames were selected (Supplementary Table S2). Frames were extracted from the trajectories using PyMOL, including the chain A protein atoms and originally coordinated Na^+^ ions. Binding pocket residues were defined as those in the 5 Å neighborhoods of the co-crystalized inhibitors in PDB 5MJU (TFB-TBOA) and 7AWM (UCPH-101). The characteristics of the different pocket conformations used for ensemble docking were further analyzed by predicting all possible pockets using the pocket finder tool in ICM-Pro and visually selecting the orthosteric and allosteric sites. Pocket volume, hydrophobicity, buriedness, and DLID score were used to confirm pocket conformational variability. ICM-Pro implementation of flexible docking (4D docking) was performed using the five extracted frames to build a receptor map complex per mutant. The rest of the docking setup and parameters followed the general framework described in section *System preparation and molecular docking*. In 4D docking, the stack of receptor conformations provided are considered as a single receptor object and ten docking poses are generated on the most favorable conformations. The best docking pose in terms of docking score for each mutant was selected for analysis.

## 3 Results

### 3.1 Cancer-related mutations are widespread across the EAAT1 structure

**FIGURE 1:**
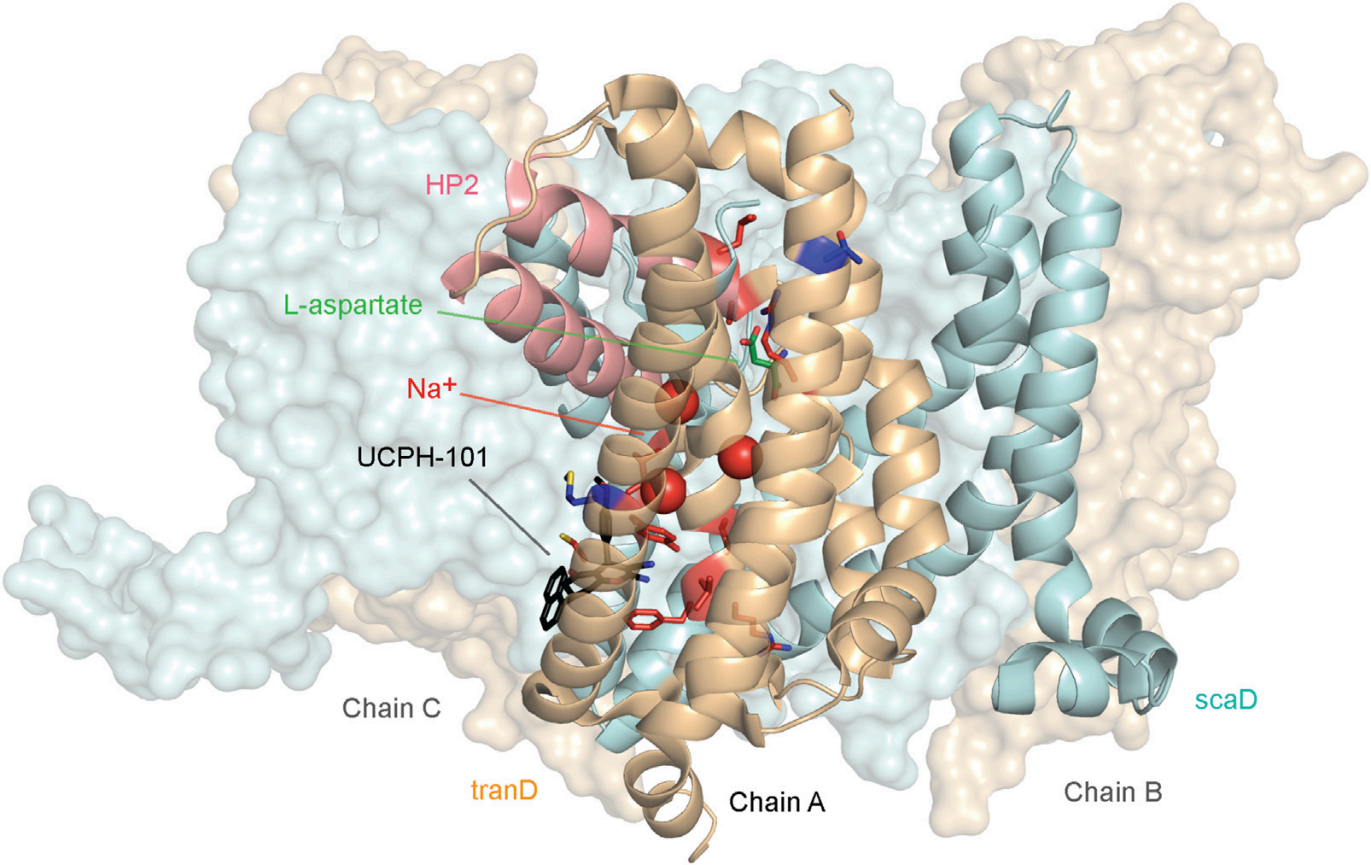
EAAT1 mutations presented in this study. Structural distribution of cancer- and ataxia-related mutants in EAAT1 functionally relevant domains presented in this study. Cancer-related mutations (Y127C, V247F, C252F, R388K, F389L, V390M, P392L, I397V, A446E, A446V, L448Q, and R479W) are mapped in red onto chain A of the EAAT1 trimer (PDB 7AWM). Ataxia-related mutations (M128R and T318A) are mapped in dark blue onto chain A. Chains B and C are represented as surfaces. Protein domains are color-coded as follows: tranD domain (orange), scaD domain (cyan), helical hairpin 2 (HP2) domain (red). The co-crystalized substrate, L-aspartate, is represented in green sticks in chain A. The three coordinated Na^+^ ions are represented as red spheres in chain A. The allosteric inhibitor UCPH-101 is represented in black sticks.

Somatic mutations in EAAT1 are found in cancer patients suffering from different cancer types. Across all cancer types in the Genomic Data Commons (GDC) (M. A. Jensen et al., 2017), 105 unique EAAT1 mutations were identified primarily located in uterine cancer (29 mutations) followed by lung cancer and melanoma (21 mutations each) and colon cancer (11 mutations). The frequency of these unique mutations is comparable to natural variance occurrence (1.18% vs 1.75%, respectively), and they are widespread across the EAAT1 structure without any specific mutational pattern observed per cancer type (Supplementary Figures S1A, B). However, most EAAT1 mutations found in cancer patients are not present in natural variance, and some of them are found in structural domains in which conformational rearrangements could lead to transport function impairment. For example, there are mutations located in the vicinity of the binding sites occupied by the substrate and coordinating Na^+^ ions, as well as in the HP2 domain (Supplementary Figures S1A, B). Moreover, certain mutations found in cancer patients are located in the binding pockets occupied by orthosteric and allosteric EAAT1 inhibitors, which could lead to changes in their binding affinity and potency. Twelve mutations not present in natural variance that were found in the functional and binding domains mentioned above (Y127C, V247F, C252F, R388K, F389L, V390M, P392L, I397V, A446E, A446V, L448Q, and R479W) were shortlisted to characterize their effect with a combination of *in silico* and *in vitro* methods (Figure 1).

### 3.2 EAAT1 mutants are predicted to have a local effect on substrate and inhibitor binding affinity

**FIGURE 2:**
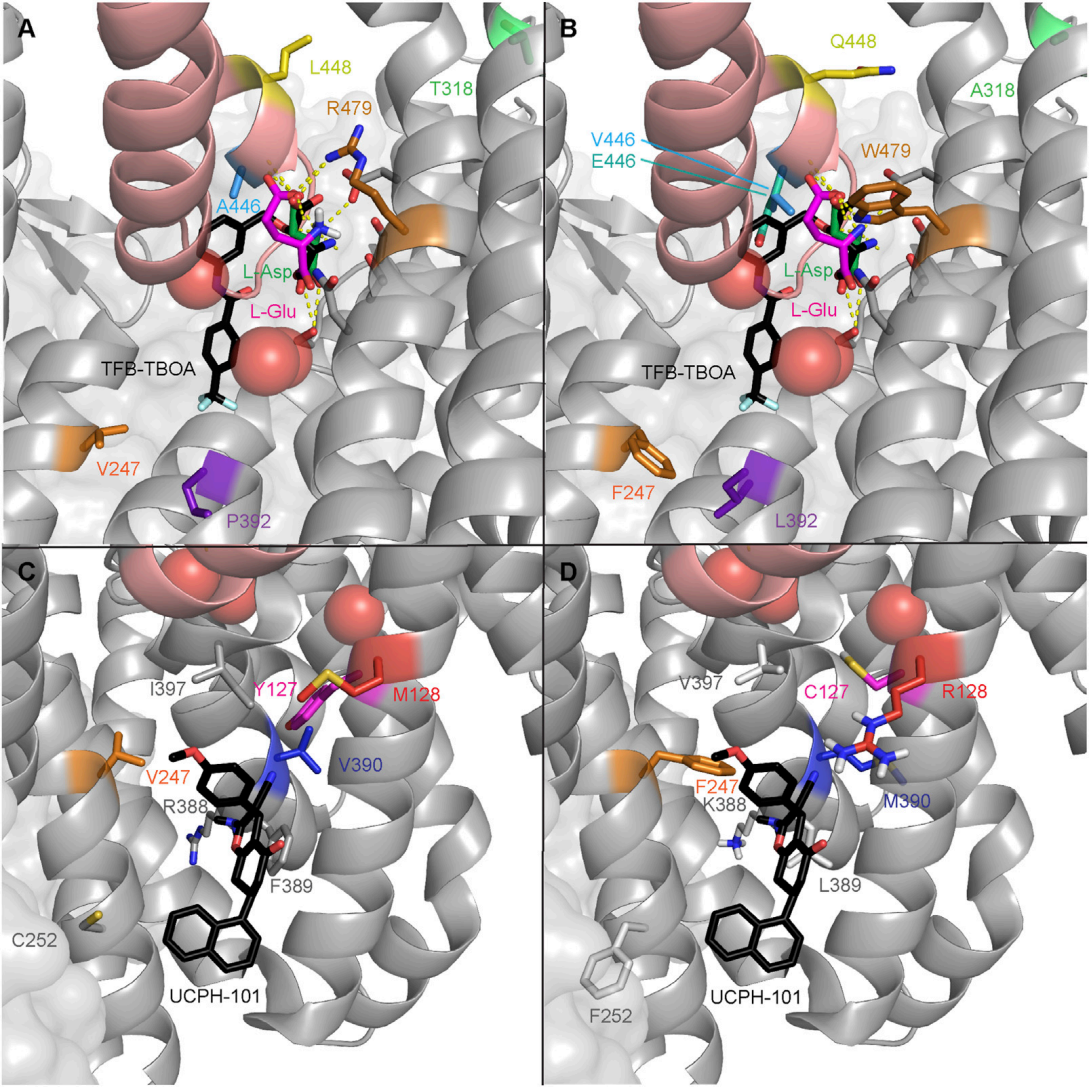
EAAT1 disease-related mutations in the orthosteric and allosteric binding sites. Mutations are mapped onto chain A of PDB 7AWM. Thermostabilizing mutations C252V and T318M were reverted in 7AWM for ΔΔG_bind_ calculation and visualization purposes. For spatial reference, the helical hairpin 2 (HP2) domain helices are colored salmon. The three coordinated Na^+^ ions are represented as red spheres. **(A)** WT residues where mutations have been found in cancer in the orthosteric binding site of EAAT1. Ataxia-related reference mutation T318A is visualized in light green. The co-crystalized substrate, L-aspartate, is represented as green sticks. The docked substrate, L-glutamate, is represented in magenta. The competitive inhibitor TFB-TBOA is represented as black sticks and superimposed to the 7AWM structure from its position in PDB 5MJU. Polar contacts between the substrate and EAAT1 are represented as dashed yellow lines. **(B)** Mutated residues in the orthosteric binding site of EAAT1. **(C)** WT residues where mutations have been found in cancer in the allosteric binding site of EAAT1. Ataxia-related reference mutation M128R is visualized in red. The co-crystalized allosteric inhibitor UCPH-101 is represented as black sticks. **(D)** Mutated residues in the allosteric binding site of EAAT1.

The effect on ligand binding affinity of cancer-related mutants found in the orthosteric and allosteric binding sites of EAAT1 was tested *in silico* to prioritize mutations for *in vitro* testing. Changes in binding energy ΔΔG_bind_ were calculated for two endogenous substrates (L-aspartate and L-glutamate), one competitive ‘orthosteric’ inhibitor (TFB-TBOA), and one non-competitive ‘allosteric’ inhibitor UCPH-101 (Table 2). Since the method employed short-range Monte Carlo sampling, the analysis was restricted to mutants in the vicinity of the ligand of interest and classified the mutants as ‘orthosteric’ (V247F, P392L, A446E, A446V, L448Q, and R479W, Figures 2A, B) and ‘allosteric’ (Y127C, V247F, C252F, R388K, F389L, V390M, and I397V, Figures 2C, D). A positive ΔΔG_bind_ over 1 kcal/mol can be interpreted as a significant decrease in binding affinity, while a negative ΔΔG_bind_ below −1 kcal/mol can be interpreted as a significant increase in binding affinity (Table 2) (Cournia et al., 2017).

**TABLE 2:** Binding energy changes (ΔΔG_bind_) predicted in ICM-Pro for EAAT1 orthosteric and allosteric mutants.^a^ ΔΔGbind was calculated for the endogenous substrates L-aspartate and L-glutamate and for the competitive inhibitor TFB-TBOA for orthosteric EAAT1 mutants. The systems used were chain A of PDB 5LLU (with L-aspartate co-crystalized and L-glutamate docked), and chain A of PDB 5MJU (with TFB-TBOA co-crystalized).^b^ For the allosteric mutants, ΔΔG_bind_ was calculated for the allosteric inhibitor UCPH-101 in Chain A of PDB 5MJU.^c^ V247F is situated between the orthosteric and allosteric site.

	Orthosteric mutants				Allosteric mutants
	ΔΔG_bind_ (kcal/mol)^a^				ΔΔG_bind_ (kcal/mol)^b^
	L-aspartate	L-glutamate	TFB-TBOA		UCPH-101
**V247F** ^**c**^	0.52	0.08	−0.70	**Y127C**	5.82
**P392L**	0.04	−0.01	−0.70	**V247F** ^**c**^	0.68
**A446E**	6.39	−0.90	1.86	**C252F**	−0.49
**A446V**	0.58	−1.73	2.23	**R388K**	−0.05
**L448Q**	−0.35	−1.88	1.79	**F389L**	3.83
**R479W**	7.13	6.42	42.19	**V390M**	−0.76
**-**	-	-	-	**I397V**	−0.62

Within the orthosteric mutants, a substantial increase in ΔΔG_bind_ values was observed in mutant R479W for both endogenous substrates and especially for the inhibitor TFB-TBOA, which indicates highly unfavorable binding of these ligands. V247F and P392L did not show significant changes as these residues are further away from the substrate’s binding site, but an incipient increased binding affinity towards TFB-TBOA was observed. A446V and L448Q, and to a lesser extent A446E, showed an increased binding affinity towards L-glutamate. Interestingly, while both A446 mutants displayed a reduced TFB-TBOA affinity, A446E and A446V showed a different profile for the two endogenous substrates. A substantial loss of binding affinity towards L-aspartate was observed in A446E, but not A446V. Within the allosteric mutants, Y127C and F389L showed a significant decrease in binding affinity towards UCPH-101. V390M showed the biggest increase in binding affinity, although this change in ΔΔG_bind_ was not significant.

Based on these results, five orthosteric (P392L, A446E, A446V, L448Q, and R479W) and two allosteric mutants (Y127C and V390M) were selected for *in vitro* testing based on their differential ΔΔG_bind_ profiles. Moreover, V247F was included in the selection since it was considered to be at the interface of both binding pockets. Of the selected residues, Y127, V390, P392, A446, L448 and R479 are fully conserved in mammalian EAATs, as well as the archaeal glutamate transporter homolog Glt_Ph_ (except V390 and L448), which suggests the relative importance of these residues in protein function (Supplementary Figure S2). To validate the *in vitro* assay, two additional EA6-associated EAAT1 mutations were selected that have been reported to either completely abolish glutamate transport (M128R) or have unaltered transport (T318A). Neither of these two residues are conserved in other glutamate transporters (Supplementary Figure S2). M128 is adjacent to Y127 and in close proximity to the binding site of UCPH-101, whereas T318 is not in the vicinity of ligand binding sites (Figure 2).

### 3.3 EAAT1 mutants respond differentially to substrates in a phenotypic assay

To assess the selected mutants for their function *in vitro*, a series of HEK293 JumpIn cell lines were generated and modified to stably express either WT (EAAT1_WT_) or mutant EAAT1 upon induction with 1 μg/mL doxycycline for 24 h. None of the ten mutants showed either a decreased or increased expression of the HA-tagged EAAT1 compared to EAAT1_WT_ after doxycycline treatment, indicating that the mutations did not affect translation of the transgene (Supplementary Figure S3).

**FIGURE 3:**
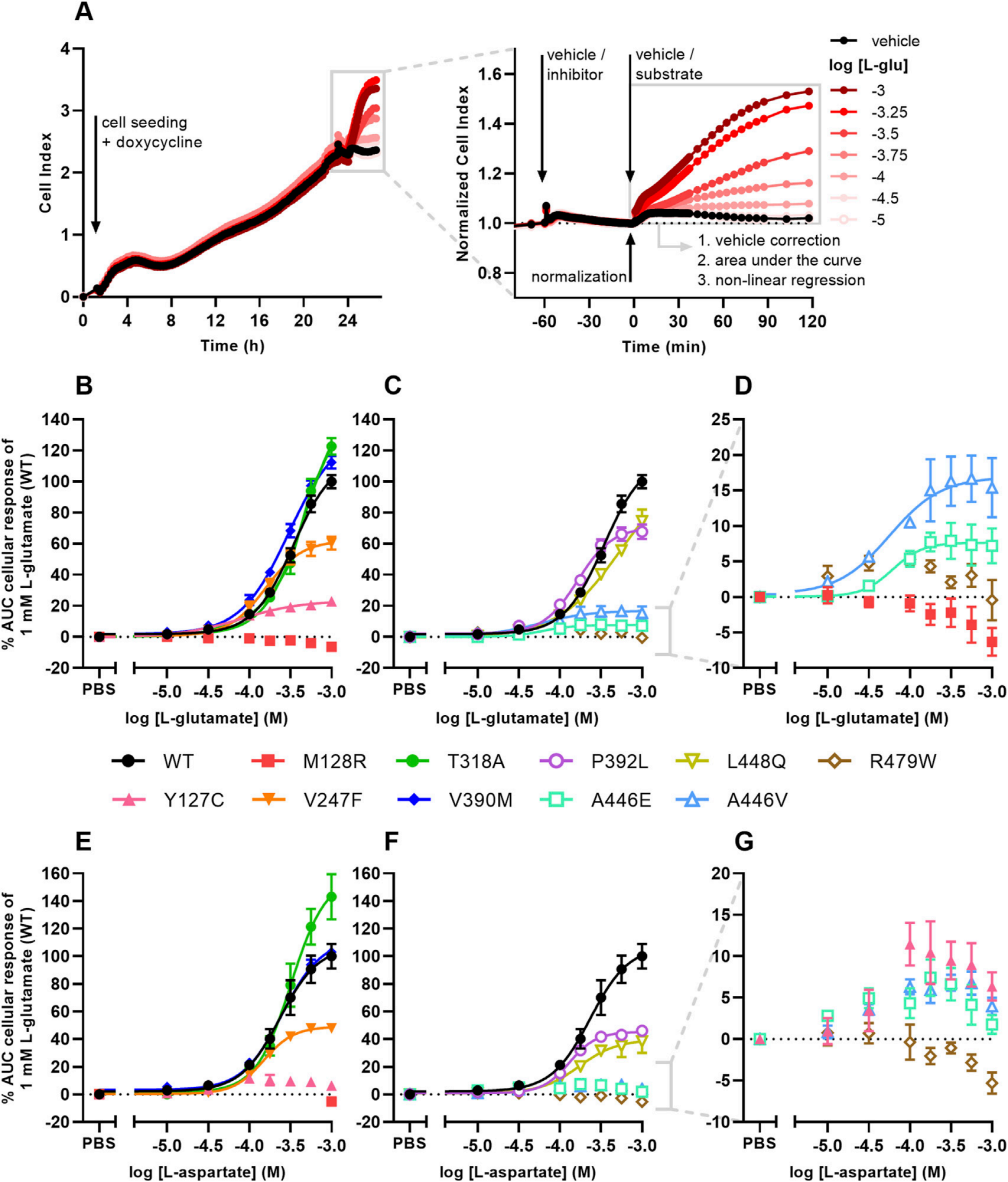
Cellular responses of L-glutamate and L-aspartate in an impedance-based phenotypic assay on EAAT1_WT_ and mutant cells. **(A)** Illustrative graph of the assay and analysis procedure. EAAT1_WT_ cells are seeded and grown for 24 h in the presence of 1 μg/mL doxycycline to induce EAAT1 expression. Cells are pretreated with vehicle (PBS/DMSO) or inhibitor (TFB-TBOA or UCPH-101, only in Figure 4) for 60 min and subsequently stimulated with vehicle (PBS) or substrate (L-glutamate or L-aspartate) for 120 min. The Cell Index (CI) is normalized prior to substrate stimulation and the cellular response is quantified by analyzing the net area under the curve (AUC). **(B–G)** Concentration-response curves of **(B–D)** L-glutamate and **(E–G)** L-aspartate on EAAT1_WT_ cells and **(B,E)** ataxia and allosteric site mutants and **(C,F)** orthosteric site mutants. **(D,G)** Zoom-in on mutants with low maximal cellular responses. Cellular response is expressed as the net AUC of the first 120 min after L-glutamate or L-aspartate stimulation. Graphs are normalized to the response of 1 mM L-glutamate or L-aspartate on EAAT1_WT_ cells. Data are shown as the mean ± SEM of three to seven individual experiments each performed in duplicate.

To assess whether the EAAT1 mutants affect transporter functionality, an impedance-based phenotypic assay was used. In this set-up, adherent cells (over)expressing EAAT1 are cultured on gold-plated electrodes in a 96-well E-plate. Upon stimulation with high concentrations (10 μM–1 mM) of substrate (i.e., L-glutamate or L-aspartate) the cells started spreading as a result of Na^+^-dependent substrate uptake via EAAT1 and subsequent cell spreading. The expanded electrode coverage by the cells generated an increase in impedance over time, which was expressed as Cell Index (CI) and interpreted as a readout of EAAT1 function (Figure 3A). Growth curves were recorded prior to inhibitor pretreatment and substrate stimulation and all mutants displayed similar CI traces compared to EAAT1_WT_, which suggested that the presence of mutant EAAT1 did not substantially affect cell adhesion or proliferation during the experiments (Supplementary Figure S4).

L-glutamate induced a concentration-dependent cellular response in EAAT1_WT_ (pEC_50_ = 3.5 ± 0.0), which was reflected by a gradual increase of the normalized Cell Index (nCI) in the first 120 min after substrate stimulation (Figures 3A–D, Table 3). A comparable L-glutamate potency was observed for the EA6 mutant T318A (pEC_50_ = 3.3 ± 0.0) with a slightly increased maximal response (E_max_), whereas the L-glutamate response was completely abolished for M128R (Figures 3B–D). The allosteric site mutants V247F (pEC_50_ = 3.8 ± 0.0) and V390M (pEC_50_ = 3.5 ± 0.0) produced similar L-glutamate potencies compared to EAAT1_WT_, where V247F has a 62% reduced E_max_ (Figure 3B). The potency of L-glutamate on Y127C was enhanced (pEC_50_ = 4.1 ± 0.1), but displayed a substantial drop (94%) in E_max_ (Figure 3B). The orthosteric site mutants P392L (pEC_50_ = 3.8 ± 0.0) and L448Q (pEC_50_ = 3.3 ± 0.1) showed no significant change in L-glutamate potency, although the concentration-effect curve for L448Q appeared more linear and shifted rightward and did not appear to reach a maximum within the tested concentration range (Figure 3C). Both A446E and A446V produced glutamate responses with a strongly reduced E_max,_ but with significantly enhanced L-glutamate potency (pEC_50_ = 4.4 ± 0.3 and 4.3 ± 0.2, respectively), whereas no concentration-dependent L-glutamate response was observed for R479W (Figures 3C, D).

**TABLE 3:** Potencies (pEC_50_) of L-glutamate and L-aspartate and inhibitory potencies (pIC_50_) of TFB-TBOA and UCPH-101 on JumpIn-EAAT1_WT_ and mutant cells in an impedance-based phenotypic assay.^a^ Maximal responses (E_max_) are normalized to the cellular response of 1 mM L-glutamate or L-aspartate (100%) on JumpIn-EAAT1_WT_ cells.

	L-glutamate		L-aspartate		TFB-TBOA	UCPH-101
	pEC_50_ (log M)	E_max_^a^ (%)	pEC_50_ (log M)	E_max_^a^ (%)	pIC_50_ (log M)	pIC_50_ (log M)
**WT**	3.5 ± 0.0	117 ± 5	3.6 ± 0.1	108 ± 9	6.7 ± 0.1	5.4 ± 0.0
**Y127C**	4.1 ± 0.1 ***	23 ± 3	N.D.	N.D.	6.2 ± 0.0 *	N.D.
**M128R**	N.D.	N.D.	N.D.	N.D.	N.D.	N.D.
**V247F**	3.8 ± 0.0	55 ± 9	3.8 ± 0.0	49 ± 1	5.7 ± 0.1 ****	5.3 ± 0.0
**T318A**	3.3 ± 0.0	156 ± 4	3.5 ± 0.0	158 ± 18	6.9 ± 0.1	5.4 ± 0.0
**V390M**	3.5 ± 0.0	132 ± 6	3.6 ± 0.0	112 ± 3	6.7 ± 0.0	5.4 ± 0.0
**P392L**	3.8 ± 0.0	71 ± 4	3.9 ± 0.0	46 ± 3	6.5 ± 0.1	N.D.
**A446E**	4.4 ± 0.3 ****	8 ± 2	N.D.	N.D.	7.4 ± 0.2 **	5.9 ± 0.2
**A446V**	4.3 ± 0.2 ****	16 ± 4	N.D.	N.D.	N.D.	N.D.
**L448Q**	3.3 ± 0.1	116 ± 25	3.7 ± 0.1	47 ± 13	7.9 ± 0.0 ****	5.9 ± 0.1 **
**R479W**	N.D.	N.D.	N.D.	N.D.	N.D.	N.D.

Next, the responsiveness of the EAAT1 mutants to the endogenous substrate L-aspartate was assessed. L-aspartate induced a concentration-dependent cellular response in EAAT1_WT_ (pEC_50_ = 3.6 ± 0.1) similar to L-glutamate (Figure 3E). The potency of L-aspartate was comparable in the EA6 mutant T318A (pEC_50_ = 3.5 ± 0.0) with an elevated E_max_, whereas in M128R no L-aspartate response was observed at 1 mM (Figure 3E). The response of L-aspartate in V390M (pEC_50_ = 3.6 ± 0.0) was identical to EAAT1_WT_ (Figure 3E). The mutants V247F (pEC_50_ = 3.8 ± 0.0), P392L (pEC_50_ = 3.9 ± 0.0) and L448Q (pEC_50_ = 3.7 ± 0.1) produced similar L-aspartate potencies, but a substantially lowered E_max_ (∼60%) compared to EAAT1_WT_ (Figures 3E, F). For Y127C, A446E and A446V the maximal L-aspartate response was reduced. Although the L-aspartate response increases at low substrate concentrations, it dropped at high concentrations, resulting in a bell-shaped concentration-effect curve from which no pEC_50_ and E_max_ were calculated (Figures 3E–G). Similar to L-glutamate, no L-aspartate response was observed for R479W (Figures 3F, G). Collectively, these data demonstrate that the selected EAAT1 mutants impact L-glutamate and L-aspartate transport.

### 3.4 EAAT1 inhibitors induce cellular response in M128R mutant

To assess whether the selected mutants modulated the effects of the competitive (‘orthosteric’) inhibitor TFB-TBOA and the non-competitive (‘allosteric’) inhibitor UCPH-101, the cells were pretreated for 1 h with increasing concentrations of inhibitor prior to stimulation with 1 mM L-glutamate. In EAAT1_WT_, inhibitor pretreatment itself did not result in substantial changes in the nCI (Supplementary Figures S5C–F). Strikingly, the M128R pretreatment with TFB-TBOA resulted in a concentration-dependent sharp nCI increase which peaked after 10–30 min, whereas pretreatment with UCPH-101 induced a more gradual nCI increase that plateaued after 60 min (Supplementary Figures S5A, B). These inhibitor responses were not observed in any of the other mutants, although V247F, A446E and A446V showed concentration-dependent decreases of the nCI upon TFB-TBOA pretreatment, which were substantially lower in magnitude compared to M128R (Supplementary Figures S5D–F). This suggests that M128R displays a distinct physiological phenotype compared to EAAT1_WT_ and other mutants.

To elucidate a potential mechanism behind the M128R response to both inhibitors, it was assessed whether the inhibitors displayed any interaction with each other or the substrate L-glutamate. Indeed, cells pretreated with TFB-TBOA were responsive to a subsequent stimulation with UCPH-101 and *vice versa*, indicating that the cellular responses elicited by either inhibitor are additive and are constituted by independent mechanisms (Supplementary Figures S6A, B). Interestingly, the response caused by TFB-TBOA pretreatment was completely blocked after stimulation with 1 mM L-glutamate and a TFB-TBOA response was prevented when cells were pretreated with L-glutamate, indicating that the TFB-TBOA response is transient and originates from interactions at the substrate binding site (Supplementary Figures S6A–C). In contrast, L-glutamate stimulation after UCPH-101 pretreatment does not reduce the nCI. The UCPH-101 response after L-glutamate pretreatment has a comparable magnitude to the UCPH-101 pretreatment on its own, suggesting that L-glutamate and UCPH-101 do not compete for the same binding site (Supplementary Figures S6B, C). In addition, the Na^+^/K^+^-ATPase (NKA) inhibitor ouabain prevented any inhibitor- or substrate-induced cellular responses in M128R cells, which indicates that TFB-TBOA and UCPH-101 responses are likely dependent on ion influx (Supplementary Figure S6D).

### 3.5 EAAT1 mutants alter TFB-TBOA and UCPH-101 inhibition

**FIGURE 4:**
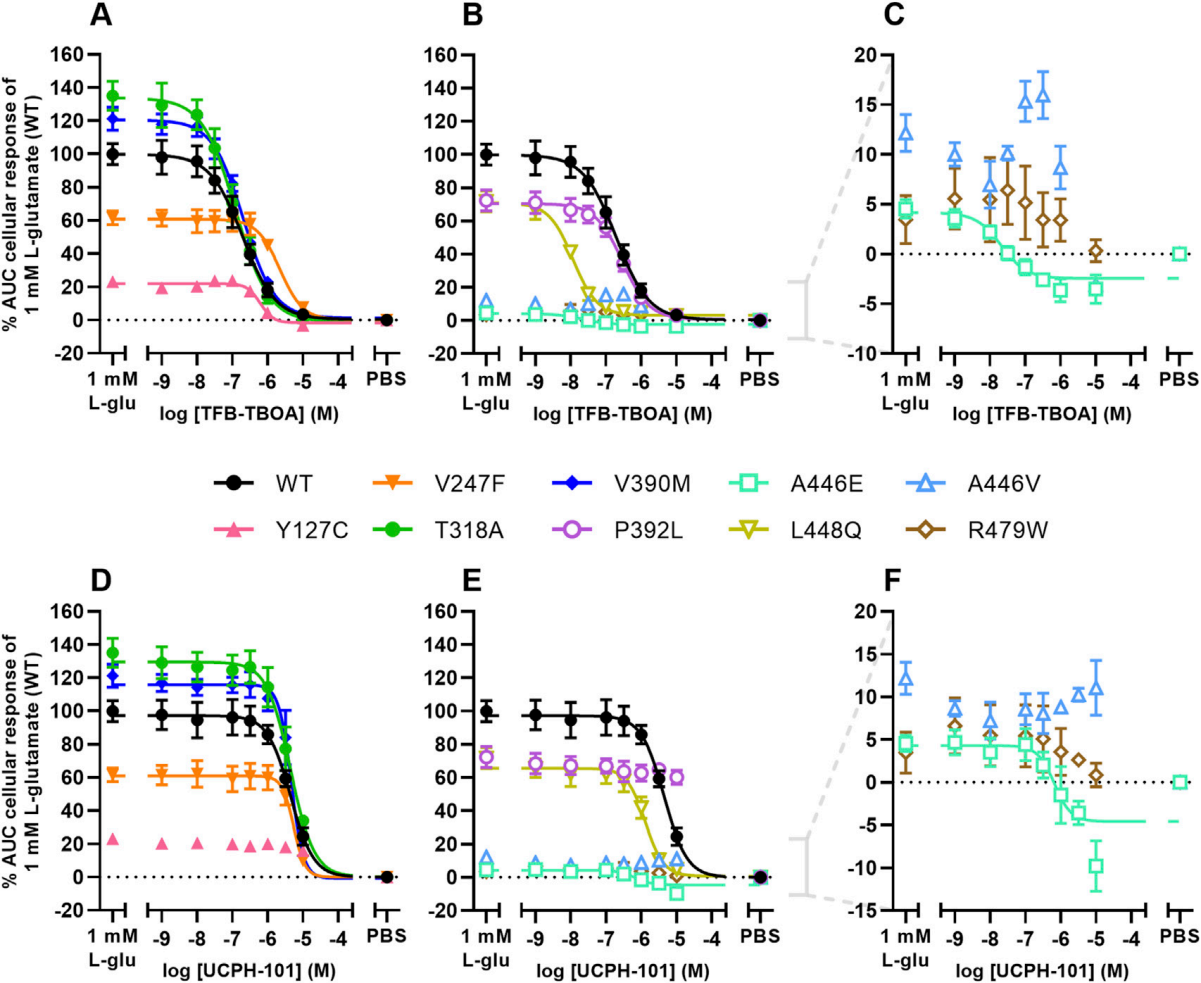
Inhibition of L-glutamate responses by TFB-TBOA and UCPH-101 in an impedance-based phenotypic assay on EAAT1_WT_ and mutant cells **(A–F)** Concentration-inhibition curves of **(A–C)** TFB-TBOA and **(D–F)** UCPH-101 on EAAT1_WT_ cells and **(A,D)** ataxia and allosteric site mutants, and **(B,E)** orthosteric site mutants. **(C,F)** Zoom-in on mutants with low maximal cellular responses. Cells were pretreated with TFB-TBOA, UCPH-101 or vehicle (PBS/DMSO) for 60 min and stimulated with a submaximal concentration (EC_80_) of 1 mM L-glutamate or vehicle (PBS) for 120 min. Cellular response is expressed as the net AUC of the first 120 min after L-glutamate stimulation and graphs are normalized to the response of 1 mM L-glutamate on EAAT1_WT_ cells. Data are shown as the mean ± SEM of three individual experiments each performed in duplicate.

For EAAT1_WT_ and all other mutants, except M128R, the inhibitory potencies of TFB-TBOA and UCPH-101 were assessed by analyzing the response of 1 mM L-glutamate after 60 min pretreatment with increasing inhibitor concentrations. In EAAT1_WT_, TFB-TBOA inhibited the L-glutamate response in a concentration-dependent manner (pIC_50_ = 6.7 ± 0.1) (Figures 4A, B; Table 3). The EA6 mutant T318A (pIC_50_ = 6.9 ± 0.1), allosteric site mutant V390M (pIC_50_ = 6.7 ± 0.0) and orthosteric site mutant P392L (pIC_50_ = 6.5 ± 0.1) did not affect the inhibitory potency of TFB-TBOA (Figures 4A, B). Both Y127C (pIC_50_ = 6.2 ± 0.0) and V247F (pIC_50_ = 5.7 ± 0.1) significantly decreased the potency, whereas L448Q (pIC_50_ = 7.9 ± 0.0) significantly enhanced the inhibitory potency of TFB-TBOA (Figures 4A, B). Interestingly, A446E was susceptible to TFB-TBOA inhibition, showing an increased inhibitory potency (pIC_50_ = 7.4 ± 0.2), whereas A446V as well as R479W did not display any sigmoidal concentration-dependent inhibition by TFB-TBOA (Figures 4B, C).

The effects of EAAT1 mutants on UCPH-101 inhibition were different from TFB-TBOA. In EAAT1_WT_, UCPH-101 could inhibit the response of L-glutamate in a concentration-dependent manner (pIC_50_ = 5.4 ± 0.0) (Figures 4D, E; Table 3). V247F (pIC_50_ = 5.3 ± 0.0), T318A (pIC_50_ = 5.4 ± 0.0) and V390M (pIC_50_ = 5.4 ± 0.0) did not affect L-glutamate response inhibition by UCPH-101 (Figure 4D). In Y127C, P932L, A446V and R479W UCPH-101 was unable to inhibit the L-glutamate response at any of the tested concentrations, indicating a loss of the UCPH-101 interaction (Figures 4D–F). Similar to TFB-TBOA, both L448Q (pIC_50_ = 5.9 ± 0.1) and A446E (pIC_50_ = 5.9 ± 0.2) enhanced the inhibitory potency of UCPH-101, although this was not significant for A446E (*p* = 0.0919) (Figures 4E, F). Taken together, these data imply that the selected EAAT1 mutants differentially modulate both substrate and EAAT1 inhibitor interactions.

### 3.6 EAAT1 mutants alter transporter conformation and substrate stability over time

**FIGURE 5:**
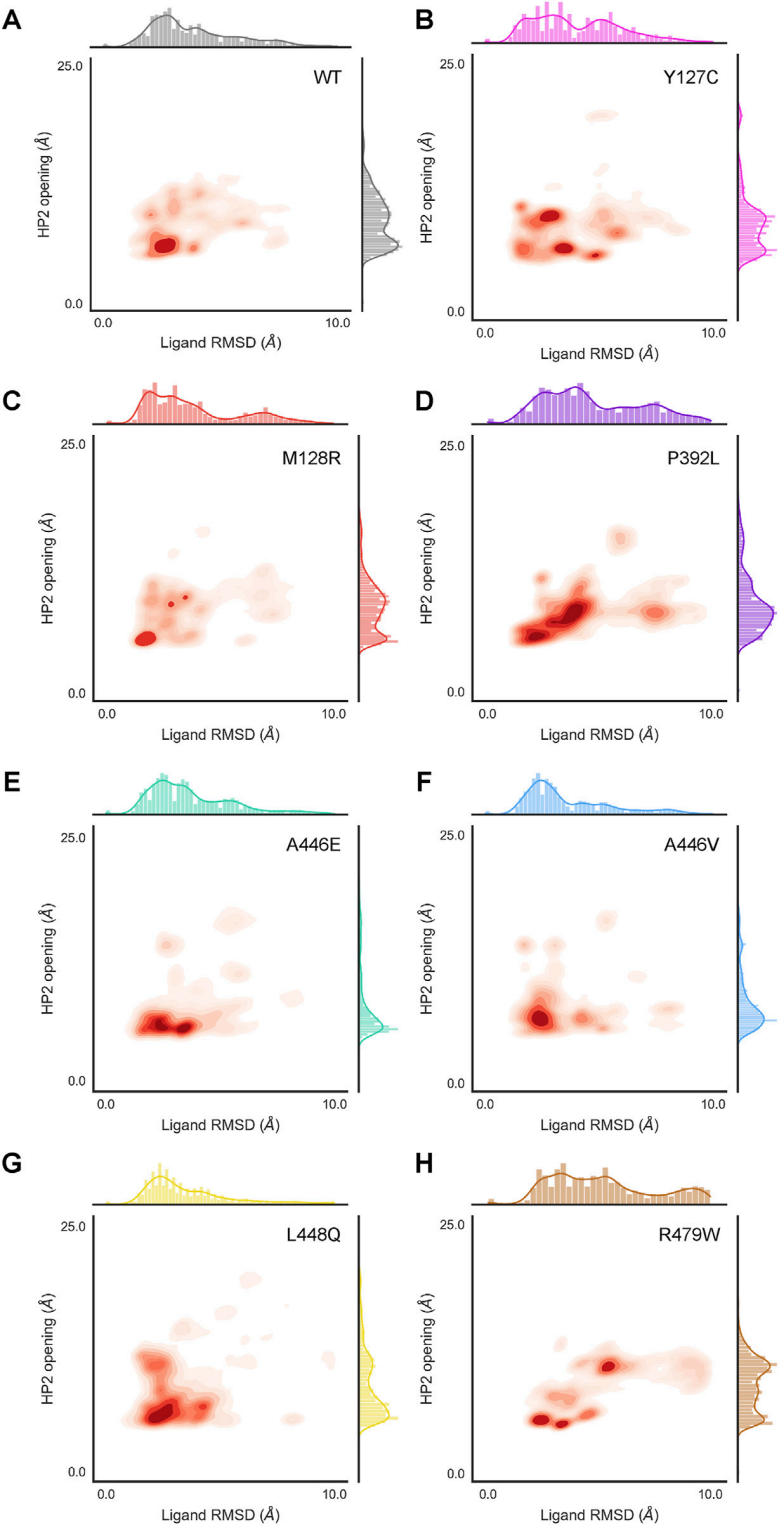
HP2 domain opening and L-Asp substrate stability sampling density derived from MD simulations on EAAT1_WT_ and mutants. HP2 opening was calculated as the distance between S366 Cα (HP1 tip) and G442 Cα (HP2 tip). Substrate (L-Asp) stability is represented by ligand RMSD respective to the protein. Sampling density was calculated across all frames in all replicates simulated for HP2 opening and substrate stability in combination (inside the axes box) and independently (outside the axes) for EAAT1_wt_ **(A)** and mutants **(B–H)**.

To assess the effect of EAAT1 mutants in transporter and substrate stability, ten replicates of 500 ns MD trajectories were simulated for the WT and seven mutants that showed differential behavior *in vitro* (Y127C, M128R, P392L, A446E, A446V, L448Q, and R479W). The simulations started from the endogenous substrate L-Aspartate-bound conformation, with coordinated Na^+^ ions in sites Na1-3 and closed HP2 domain. This represents the transporter conformation prior to its transition to the inward facing conformation. The stability of this conformation was followed over time in regards to the system overall (i.e., protein RMSD), the substrate in the binding site (i.e., ligand RMSD in respect to protein), opening of the HP2 domain (i.e., distance between the HP1 and HP2 domain tips), and coordination of the Na^+^ ions (i.e., distance between Na^+^ ion and one coordinating atom). Compared to WT (Figure 5A), mutants A446E, A446V, and L448Q (Figures 5E–G) showed a similar high ligand stability (i.e., low ligand RMSD), which correlated with a stabilized ‘closed’ HP2 conformation. HP2 domain closure was especially pronounced in A446E and A446V mutants compared to WT. On the contrary, ligand instability was higher in mutants Y127C, P392L, and R479W (Figures 5B,D,H), which correlated with increased opening of the HP2 domain, particularly in R479W. In R479W, substrate instability was also directly linked to the loss of key interactions of L-aspartate in the binding pocket, mainly with R479 and T402 (Supplementary Figures S7, S8). Mutant M128R (Figure 5C) showed a very similar distribution to WT both in terms of HP2 opening and ligand stability, which suggests that the mutation in M128 does not directly affect the conformation of the orthosteric binding site.

While the mutant effects on transporter conformation (i.e., HP2 opening) affected ligand stability, they barely had an impact on Na^+^ ion coordination. Firstly, from the MD simulations it was observed that the Na^+^ ions coordinated in sites Na1 and Na3 were extremely stable in the WT system and all mutants simulated (Supplementary Figures S9A, H). In particular, mutant M128R seemed to heavily restrict movement for the Na^+^ ion coordinated in position Na3 compared to the rest of mutants (Supplementary Figure S9C). On the contrary, the ion occupying site Na2, which is coordinated in the last place before HP2 closure, was highly unstable across the board (Supplementary Figures S9I, P). Compared to WT, Na2 was more unstable in mutants A446V and L448Q (Supplementary Figures S9N, O). However, Na^+^ coordination instability in Na2 site was not correlated to HP2 opening, since ion instability was observed both at lower and higher HP2 opening distances.

### 3.7 EAAT1 mutant-driven conformational changes impact inhibitor docking binding poses

**FIGURE 6:**
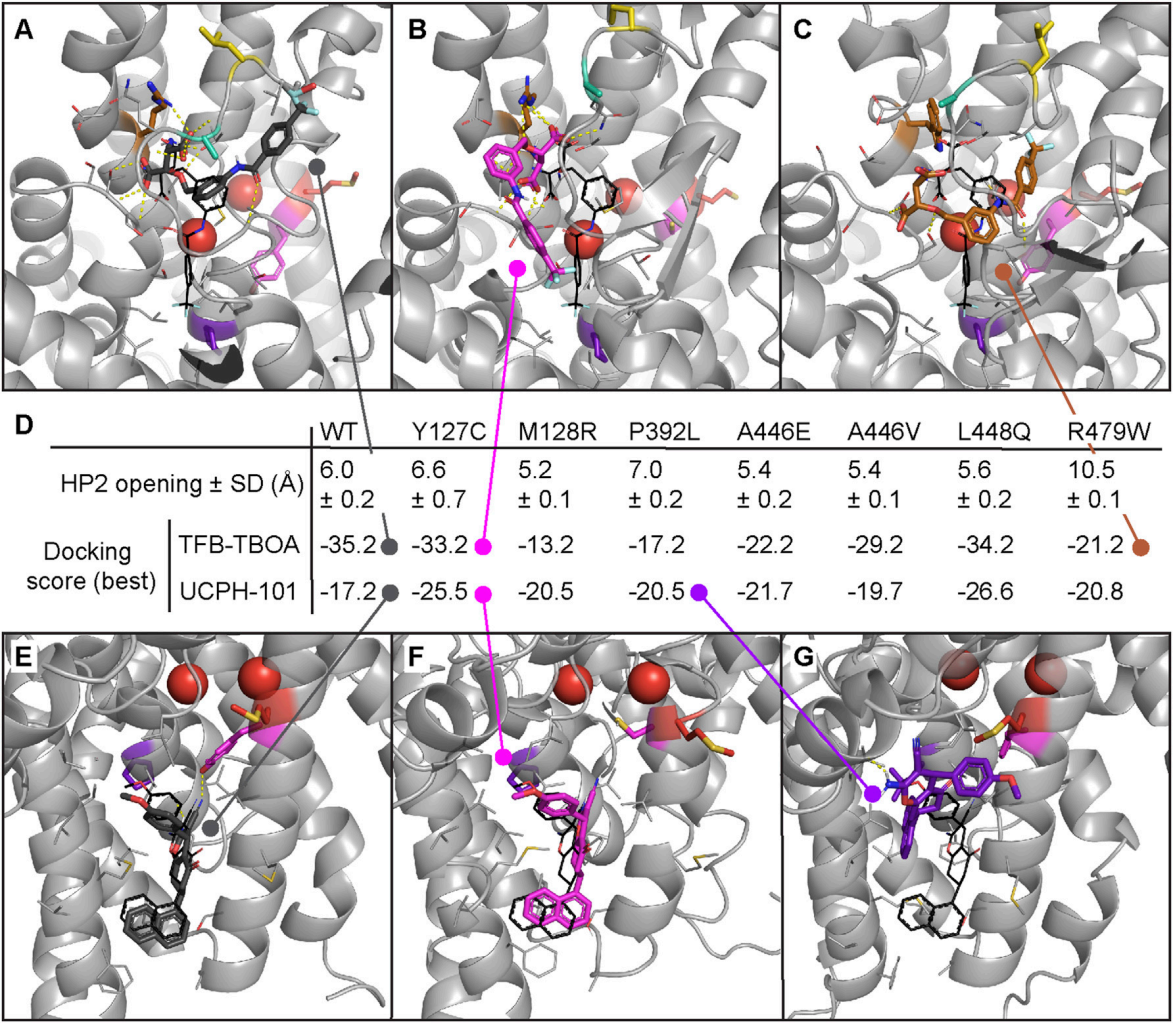
Molecular docking of inhibitors TFB-TBOA and UCPH-101 in EAAT1 MD frames with most representative HP2 opening distances. Docking performed in chain A of a random selection of frames with the top five most common HP2 opening distances across all replicates and frames **(A–C)** Top docking poses of orthosteric inhibitor TFB-TBOA in EAAT1_WT_. **(A)** And mutants Y127C **(B)** and R479W **(C)**. TFB-TBOA binding pocket was derived from its co-crystalized pose in PDB 5MJU, represented in black for reference. **(D)** Mean HP2 opening distance in the five frames selected from MD for docking. Docking scores of the top poses in EAAT1_WT_ and mutants. **(E–G)** Top docking poses of allosteric inhibitor UCPH-101 in EAAT1_WT_ **(E)** and mutants Y127C **(F)** and P392L **(G)**. UCPH-101 binding pocket was derived from its co-crystalized pose in PDB 7AWM, represented in black for reference. Na^+^ ions are represented as red spheres. Hydrogen bonds represented with dashed yellow lines.

To evaluate whether the conformational changes in the HP2 domain observed upon mutation affect inhibitor binding as they do substrate coordination, molecular docking was performed per mutant in a representative selection of five frames from the MD trajectories (Figure 6). The selected frames represented the most common HP2 opening distances per mutant: 6.0 ± 0.2 Å (WT), 6.6 ± 0.7 Å (Y127C), 5.2 ± 0.1 Å (M128R), 7.0 ± 0.2 Å (P392L), 5.4 ± 0.2 Å (A446E), 5.4 ± 0.1 Å (A446V), 5.6 ± 0.2 Å (L448Q), and 10.5 ± 0.1 Å (R479W), but had different orthosteric and allosteric pocket conformations (Supplementary Tables S3, S4). The highest scoring poses in TFB-TBOA docking roughly maintained the position and polar interactions of the aspartic acid moiety observed in the co-crystalized conformation (Supplementary Figure S10). The rest of the molecule, however, could be flipped around the two contiguous chiral centers to different positions depending on the exact conformation of the HP2 domain. This behavior was observed for the WT (Figure 6A) and mutants Y127C (Figure 6B), A446V (Supplementary Figure S10G), and L448Q (Supplementary Figure S10H). The lower scoring pose on mutant A446E (Supplementary Figure S10F) also maintained the aspartic acid moiety position, but the rest of the molecule was forced into a less stable conformation due to the HP2 configuration induced by E446 interactions. None of the lowest scoring poses in mutants M128R, P392L, and R479W maintained the aspartic acid moiety position. In mutant R479W (Figure 6C) this effect was due to the less flexible and bulkier side chain of W479, which pushed TFB-TBOA deeper in the pocket causing the loss of key interactions (Supplementary Figures S7, S8).

Compared to TFB-TBOA, the binding of allosteric inhibitor UCPH-101 was less affected by mutations as represented by the range in docking scores (Figure 6D) and poses (Supplementary Figure S11). The pose observed in co-crystalized structures was maintained in the top docking poses in WT (Figure 6E) and mutants Y127C (Figure 6F) and L448Q (Supplementary Figure S11H). The top poses in mutants Y127C and L448Q also showed a higher docking score (−25.5 and −26.6, respectively) compared to WT (−17.2), although only the pose on L448Q maintained one of the two hydrogen bonds in the co-crystalized pose to P389. UCPH-101 docked in mutant A446E (Supplementary Figure S11F) occupied the same region but the pose was flipped compared to WT. Docking poses in mutants M128R, P392L, A446V, and R479W (Supplementary Figures S11D,E,G,I) did not reach the allosteric pocket deeply enough to make relevant interactions. In the case of mutants M128R and R479W, there seemed to be a closure of the binding pocket entrance flanked by TM4c (ScaD) and TM3 (TranD). For P392L, the lower part of the pocket seemed not accessible based on the best docking pose (Figure 6G). The mutation to Leu in P392 reverted the helix kink that was produced by Pro in that position in the TM7a domain and that stabilized the allosteric binding pocket (Supplementary Figure S12). Taken together, these results suggest that EAAT1 conformational changes triggered by disease-related mutations affect the way inhibitors TFB-TBOA and UCPH-101 bind to the orthosteric and allosteric pockets, respectively.

## 4 Discussion

The role of glutamate and aspartate in cancer is increasingly appreciated (Vettore et al., 2020). Indeed, the regulation of intra- and extracellular levels of these amino acids by EAATs and other transporters, in respect to the tumor microenvironment, is the subject of ongoing investigations. So far, altered function of EAAT1 as a result of single missense mutations has been linked to several extremely rare cases of episodic ataxia type 6 (EA6) (Chivukula et al., 2020). However, there have been no reports on the contribution of genetic variants of EAATs to the development of cancer, and it remains a question to what degree loss- or gain-of-function mutations in these transporters are relevant for disease progression. In this study 105 unique somatic mutations were identified in cancer patients, none of which occurred as natural variants. Eight cancer-associated and two reference EA6-related EAAT1 missense mutants were analyzed in a label-free phenotypic assay, which together with structural insights provides an initial understanding of altered transporter function and cell behavior.

All EAAT1 mutants were expressed at similar relative levels compared to EAAT1_WT_, therefore not affecting protein translation (Supplementary Figure S3). Interestingly, in previous studies several EAAT1 mutants displayed attenuated or increased glutamate uptake activity as a result of reduced (P290R, M128R (Winter et al., 2012; Chivukula et al., 2020)) or enhanced (E219D, T318A (Adamczyk et al., 2011; Chivukula et al., 2020)) surface membrane density, respectively. Indeed, in our functional assay T318A showed a considerable increase in substrate E_max_ (Figures 3B, E; Table 3), which may be attributed to an enhanced membrane insertion of EAAT1 (Chivukula et al., 2020). Most other mutants displayed a substantial decrease in substrate E_max_, with the maximal response being generally lower for L-aspartate than L-glutamate.

Tyr at position 127 is located in TM3 and is conserved in all human EAATs and the archaeal Glt_Ph_ (Supplementary Figure S2), where the backbone carboxylate of Tyr is part of the third Na^+^ binding site (Na3) (Alleva et al., 2020; Qiu et al., 2021; Canul-Tec et al., 2022). Substitution of Y127 to Cys does not affect the ability of EAAT1 to translocate substrate, albeit with a substantially reduced E_max_ (Figure 3B). In addition to forming Na3, Y127 forms a hydrogen bond with the carbonitrile group of UCPH-101 (Canul-Tec et al., 2017). The docking studies suggest that this bond cannot form in Y127C (Figure 6F). However, the Y127C mutation seems to lead to an opening of the TM3 helix and widening of the tranD-scaD interface pocket that makes it less suitable for blocking the elevator mechanism, which might be related to the loss of UCPH-101 inhibition (Figure 4D). In line with this, mutation of Y127 to Phe, Leu, Ile or Arg showed a significant drop in pIC_50_ of UCPH-101 in a [^3^H]-D-aspartate uptake assay (Abrahamsen et al., 2013).

M128 is adjacent to Y127 and is exposed to membrane lipids. The M128R mutation was found in an EA6 patient and patch clamp experiments demonstrated that M128R shows a complete loss of glutamate uptake as well as abolished anion currents that could not be explained by slightly reduced surface expression levels (Chivukula et al., 2020). Indeed, no L-glutamate or L-aspartate responses in M128R (Figures 3B, E) were detected, which suggests that this mutant is likely transport incompetent. Surprisingly, substantial concentration-dependent positive cellular responses were observed when M128R cells were treated with TFB-TBOA or UCPH-101, which were not observed in EAAT1_WT_ or other mutants (Supplementary Figure S5). Although our computational studies did not shed any light into the potential mechanism of the observed behavior (Figure 5C, Supplementary Figures S9C, K), a recent study demonstrated that mutation of M128 to Arg may inflict two potential disruptions to EAAT1 (Wu et al., 2022). The positively charged Arg could flip towards the ‘inside’ of the protein and disrupt the binding of Na^+^ to Na3. Occupation of this site by Na^+^ is crucial to initiate substrate binding and translocation (Bastug et al., 2012), which may explain the absence of glutamate transport in M128R. In our simulations, however, a tighter coordination in Na3 was observed. Secondly, the Arg in M128R could flip ‘outward’ towards the lipid bilayer. Other MD studies revealed a local membrane deformation, which recruited a density of water molecules halfway into the bilayer (Wu et al., 2022). This may provide a pathway for Na^+^ ions that enter the Na3 site to leak into the cytosol, which could result in cell volume increase and subsequent morphological changes (Sijben et al., 2022). Thus, we hypothesize that binding of TFB-TBOA or UCPH-101 to EAAT1 M128R stabilizes an Arg ‘outward’ conformation that allows uncoupled Na^+^ influx, which results in a phenotypic response in the absence of substrate (Supplementary Figures S5, S6). To our knowledge, this is the first report of inhibitor-induced functional responses in glutamate transporters, which warrants further investigation and could hold promise for future therapeutic strategies.

The second episodic ataxia-derived mutant, T318A, showed no signs of affecting EAAT1 transporter function other than an increased substrate E_max_, in line with the lack of evidence of its pathogenicity (Choi et al., 2017; Wu et al., 2022). In other studies, mutation to Ala increased glutamate uptake and anion currents as a result of increased surface expression of the transporter (Chivukula et al., 2020; Wu et al., 2022). A similar conservative effect was found for both Val 247 and 390, which are located adjacently to hydrophobic residues conferring the selectivity of UCPH-101 towards EAAT1 (Canul-Tec et al., 2017). However, mutations V247F and V390M did not affect substrate translocation (Figure 3) or UCPH-101 binding (Figure 4D), indicating that these residues are not crucial for inhibitor binding. Interestingly, TFB-TBOA’s inhibitory potency was reduced in V247F (Figure 3), possibly due to the increased residue bulkiness affecting the hydrophobic cavity size.

The Pro at position 392 is located in TM7a near V390 and is completely conserved throughout the SLC1 family and Glt_Ph_ (Canul-Tec et al., 2017). P392 is part of the scaD–tranD interface that lines the hydrophobic cavity of the chloride conductive pathway (Seal and Amara, 1998; Chen et al., 2021). Mutation of P392 to small hydrophobic residues (Ala, Val) resulted in slightly increased substrate affinities and anion conductances (Cater et al., 2014), which may be reflected by a small increase in pEC_50_ for L-glutamate and L-aspartate in P392L (Table 3). Strikingly, while TFB-TBOA binding is unaffected, P392L causes a complete loss of UCPH-101s inhibition of the L-glutamate response (Figures 4B, E; Table 3). As observed in MD simulations, mutation to a slightly bulkier Leu corrects the disruption in the helical turn caused by Pro in TM7a (Supplementary Figure S12) and promotes an increase in helix rigidity that displaces the location of the nonpolar residues in this region. This substantially reduces the affinity of UCPH-101 for this site, as observed by the loss of the original binding pose in the docking results (Figure 6G). Interestingly, other EAAT1 Pro mutations have been shown not to revert the kink, as opposed to original hypotheses (Colucci et al., 2023).

Three mutations (A446E, A446V and L448Q) are located in HP2, which is an important structural element that regulates the access of Na^+^ and substrate to their binding sites (Boudker et al., 2007; Canul-Tec et al., 2017). In our phenotypic assay both A446E and A446V displayed vastly reduced maximal substrate responses but significantly increased affinities (Table 3), which could be the result of low surface expression or a reduced turnover rate (Trinco et al., 2021). Tracking the HP2 opening over time suggests that mutations in the HP2 domain increase the stability of a ‘closed’ conformation in the presence of bound L-Asp compared to WT (Figures 5E–G). Such ‘closed’ conformation could be the result of tighter interactions with the endogenous substrate and lead to reduced transport rate (Ciftci et al., 2021). Notably, mutation to Val at this position abrogates L-glutamate response inhibition, whereas a Glu substitution results in a significantly enhanced potency of TFB-TBOA (Figures 4C, F; Table 3). The stabilization of a ‘closed’ HP2 conformation might reduce access to the orthosteric pocket for competitive inhibitors such as TFB-TBOA or, alternatively, induce a higher inhibitory potency by locking in place the aspartic acid moiety (Canul-Tec et al., 2017). The differential effects observed for mutants A446E and A446V, however, cannot be explained by the current *in silico* studies, where a more favorable TFB-TBOA binding pose is predicted for A446V compared to A446E (Supplementary Figures S10F, G). A clear hindrance here is docking the orthosteric inhibitor in a marked HP2 ‘closed’ conformation, when TFB-TBOA is known to stabilize an ‘open’ HP2 conformation in the transporter (Canul-Tec et al., 2017).

The adjacent HP2 residue L448 is involved in HP2 backbone flexibility, which is essential for K^+^-dependent re-translocation of the tranD during the transport cycle (Kortzak et al., 2019). Strikingly, the pIC_50_ for both TFB-TBOA and UCPH-101 are markedly increased in L448Q. These results are also supported by the favorable poses generated from the docking studies for both inhibitors (Supplementary Figures S10H, S11H). In a previous study, mutation of L448 to Cys reduced L-glutamate affinity and maximal transport rate, but it significantly enhanced the inhibitory potency of the competitive inhibitor DL-TBOA (Leighton et al., 2002). The enhanced pIC_50_ for both UCPH-101 and TFB-TBOA may be the result of a reduced affinity of L-glutamate in the orthosteric site, which could augment the apparent inhibitory potency.

The Arg at position 479 confers substrate selectivity and is conserved among glutamate/aspartate transporters. The guanidinium group of R479 forms a hydrogen bond with the sidechain carboxylate of the substrate during translocation (Canul-Tec et al., 2017). Moreover, R479 forms a salt bridge with E406 in TM7 during K^+^ re-translocation, which sterically hinders closure of HP2 and substrate binding (Kortzak et al., 2019; Canul-Tec et al., 2022). Neutralization of R479 (i.e., mutation to Ala) renders EAAT1 K^+^-independent and results in drastically reduced glutamate/aspartate affinity (Kortzak et al., 2019), which was also observed in Glt_Ph_ upon mutation of Arg to Cys (Scopelliti et al., 2018). As observed in MD simulations, the bulkiness of the indole moiety pushes the HP2 domain to an ‘open’ conformation (Figure 5H) and disrupts the electrostatic interactions in the binding site (Supplementary Figure S8), which leads to a loss of substrate activity (Figure 2B). This local effect was already evident from the relatively high ΔΔG_bind_ values for R479W compared to other mutated residues (Table 2), which indicate a substantially reduced ligand binding affinity.

Discrepancies observed between the *in vitro* and *in silico* experiments likely arise from the fact that the simulations focused only on a small part of the complex elevator transport cycle and cannot therefore provide a complete mechanism for all the analyzed mutants. Adding to the complexity of the system, heterogeneity was observed among the dynamic behavior of the three protomers, which has been described for glutamate transporter analogs to trigger heterogeneous substrate binding (Reddy et al., 2022). These results warrant follow-up *in vitro* or *in silico* experiments that investigate alterations in protein solvation, anion conductivity and substrate transport kinetics (Wang et al., 2021; Wu et al., 2022), which could help to further explain our functional observations. Moreover, while mutations in a ligand binding site may disrupt or stabilize ligand interactions, they could potentially lead to allosteric effects via disruption of conserved interaction networks (Levine et al., 2016).

Taken together, divergent effects of EAAT1 disease-related variants were observed on substrate-induced cellular responses, as well as orthosteric and allosteric inhibition, in an impedance-based phenotypic assay. Subsequent MD simulations and docking studies aided in the formulation of hypotheses that could substantiate the observed *in vitro* effects. Importantly, to allocate these missense variants to a substantial involvement in cancer development and progression translational studies that link genotype to phenotype would be required. Thus, the methods presented in this study may aid in the identification and characterization of pathogenic transporter variants, which may have implications for the development of selective and efficacious therapeutics.

## Data Availability

The datasets analyzed for this study can be found in online repositories. The cancer mutations dataset can be found in the 4TU repository (https://doi.org/10.4121/15022410.v1). The 1000 Genomes dataset is available online at the Uniprot database (https://www.uniprot.org/). All protein structures used in this work are available on the RCSB Protein Data Bank (https://www.rcsb.org/). All other data presented in this study are included in the article and Supplementary Material.
