# Supplementary material for: Molecular insights into disease-associated glutamate transporter (EAAT1 / SLC1A3) variants using in silico and in vitro approaches
Source: Front Mol Biosci. 2023 Nov 23;10:1286673. doi: 10.3389/fmolb.2023.1286673 (PMC10702391; doi:10.3389/fmolb.2023.1286673)
Supplement: Supplementary file 1 [file DataSheet1.docx]

Supplementary Material


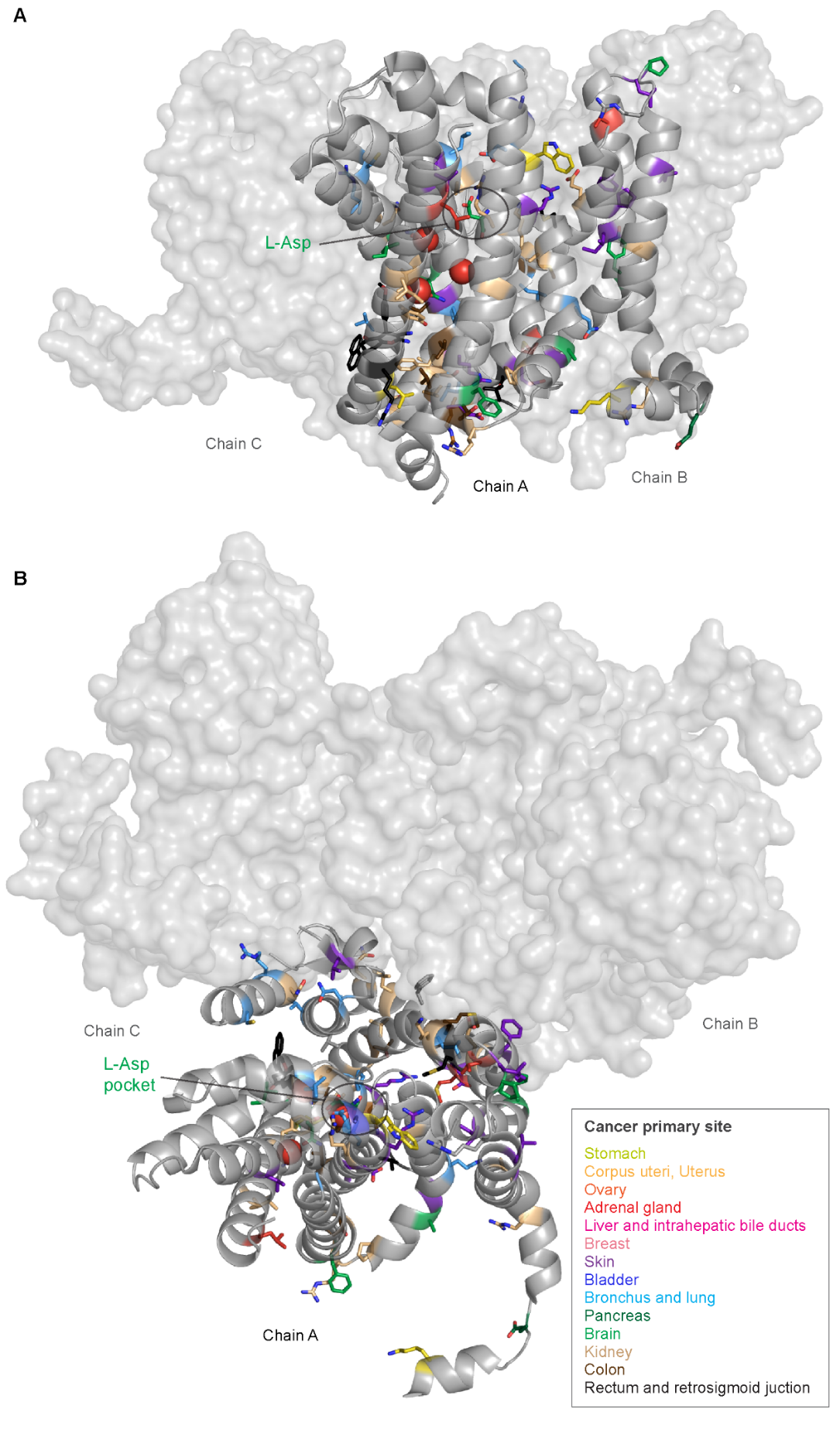


**Supplementary Figure 1**. Structural distribution of cancer-related mutants per cancer type. Mutations from the Genomic Data Commons mapped onto the biological assembly of EAAT1 (PDB 7AWM). Chain A is represented as a grey cartoon, while chains B and C are represented as grey surfaces. The co-crystalized substrate, L-aspartate, is represented as green sticks in chain A. The three coordinated Na+ ions are represented as red spheres in chain A. Residues that have been observed mutated in cancer patients are colored by cancer primary site following the colors in the key. **(A)** Frontal view, as aligned with cellular membrane. **(B)** Top view, as seen from the extracellular side.


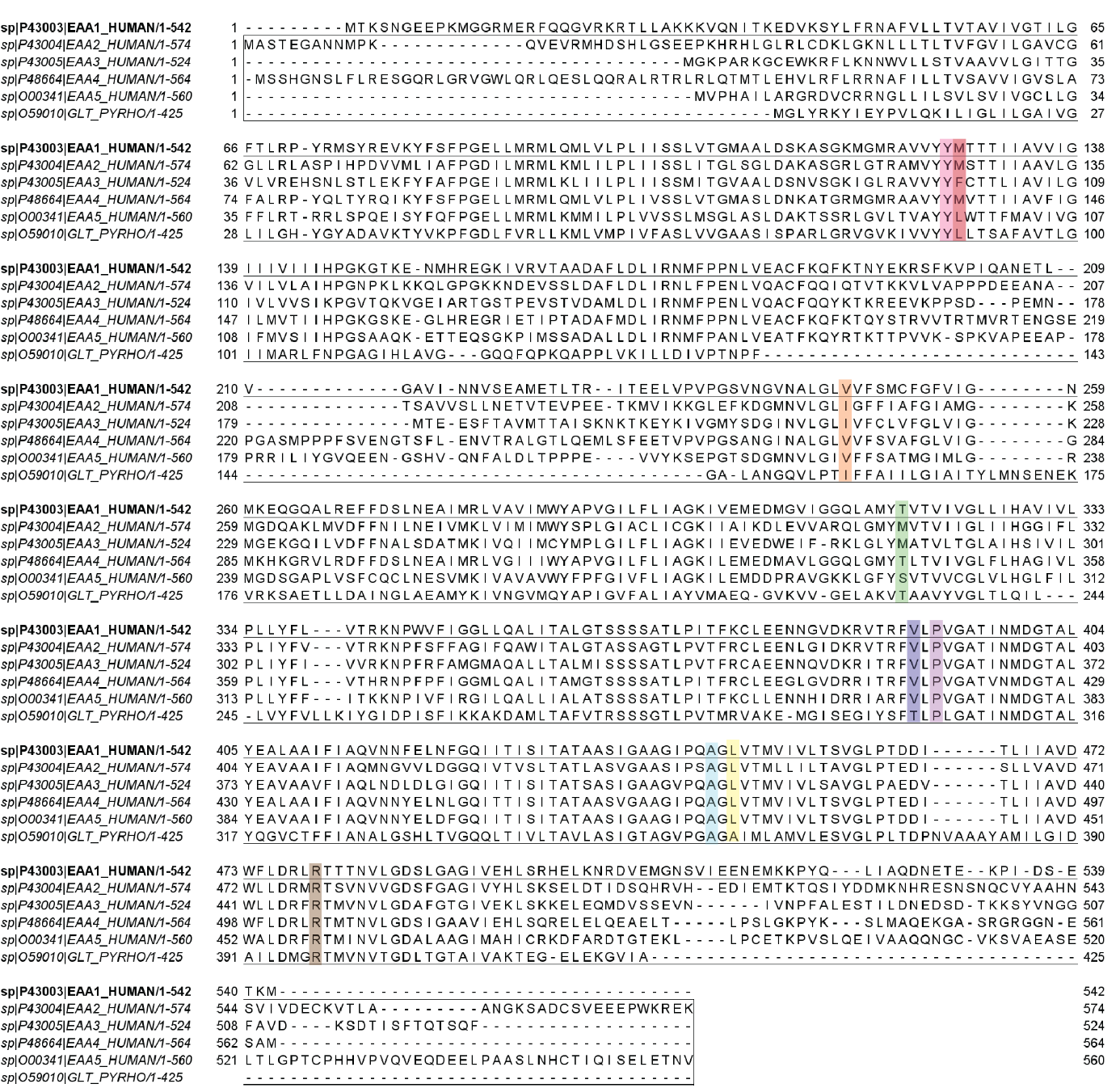


**Supplementary Figure 2. Conservation of selected cancer-related mutants in EAAT family.** Multiple sequence alignment of human EAATs (EAAT1-5) and *Pyrococcus horikoshii* homolog Glt_Ph_ computed in Clustal-Omega. Colored, the positions of the cancer-related mutants analyzed *in vitro:* Y127C (pink), V247F (orange), V390M (dark blue), P392L (purple), A446V/E (blue), L448Q (yellow), R479W (brown). For reference, ataxia-related reference mutants are also colored: M128R (red) and T318A (green).


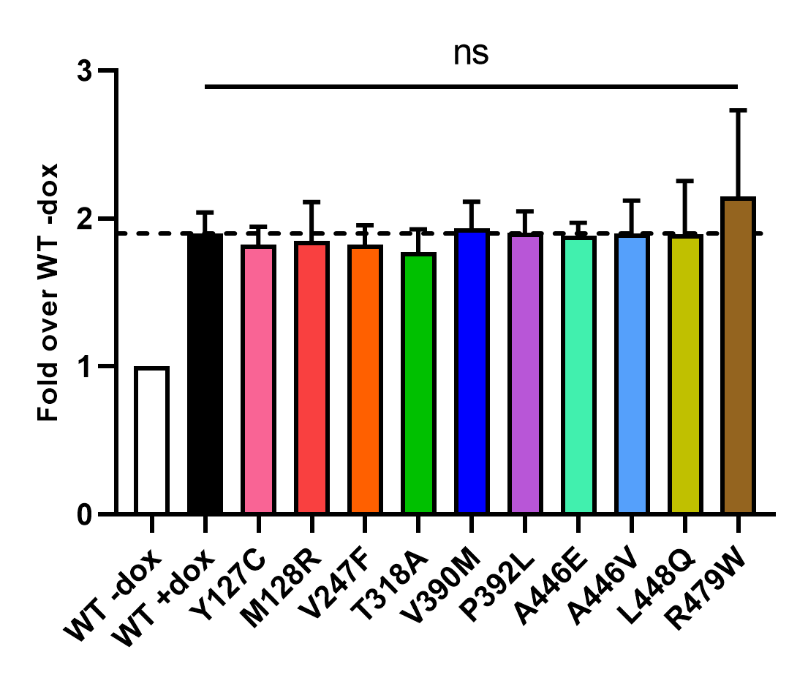


**Supplementary Figure 3.** Whole cell HA-tag ELISA on EAAT1_WT_ and mutant cells. Cells were grown for 24 h in the absence (-dox, WT only ) or presence (+dox, WT and mutants) of 1 µg/ml doxycycline. Presence of total HA-tagged protein (plasma membrane and cytosolic) was determined in permeabilized cells. Absorbance for each condition is expressed as fold expression over WT (-dox). Data are shown as the mean ± SEM of twelve (WT), six (M128R) or three (rest) individual experiments each performed in quintuplicate. Significant differences between EAAT1_WT_ and mutant cells was determined using one-way ANOVA with Dunnett’s post-hoc test. ns = not significant for all mutants.


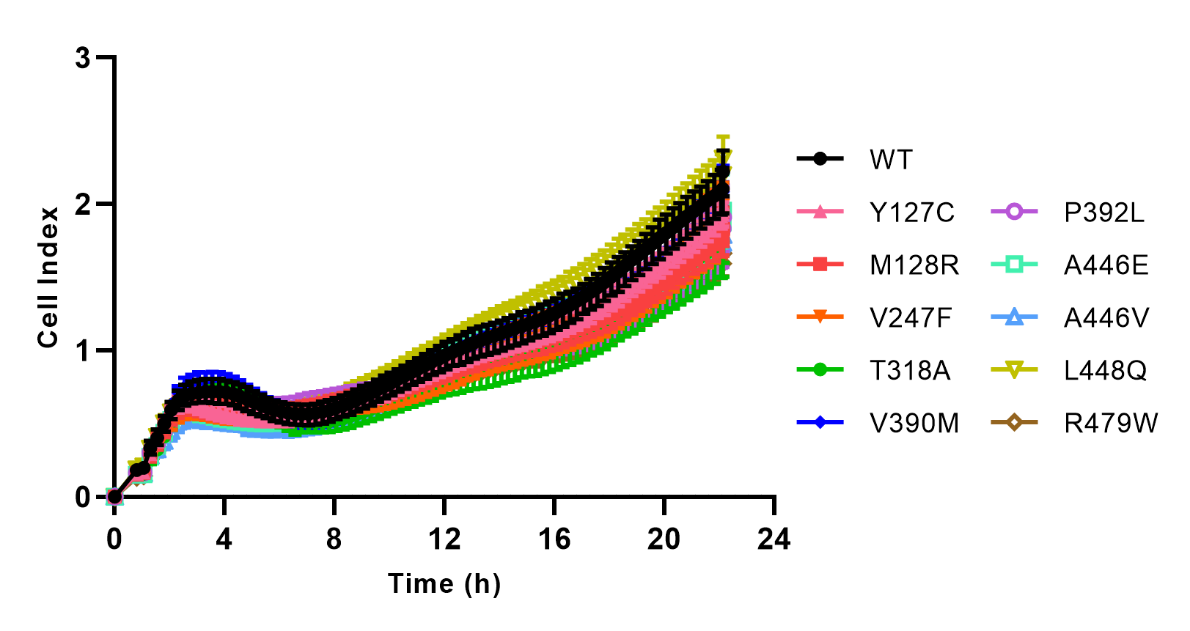


**Supplementary Figure 4**. Representative growth curves of EAAT1_WT_ and EAAT1 mutant cells in an impedance-based phenotypic assay. Data are shown as the mean ± SD of eight replicates from a representative experiment.

**
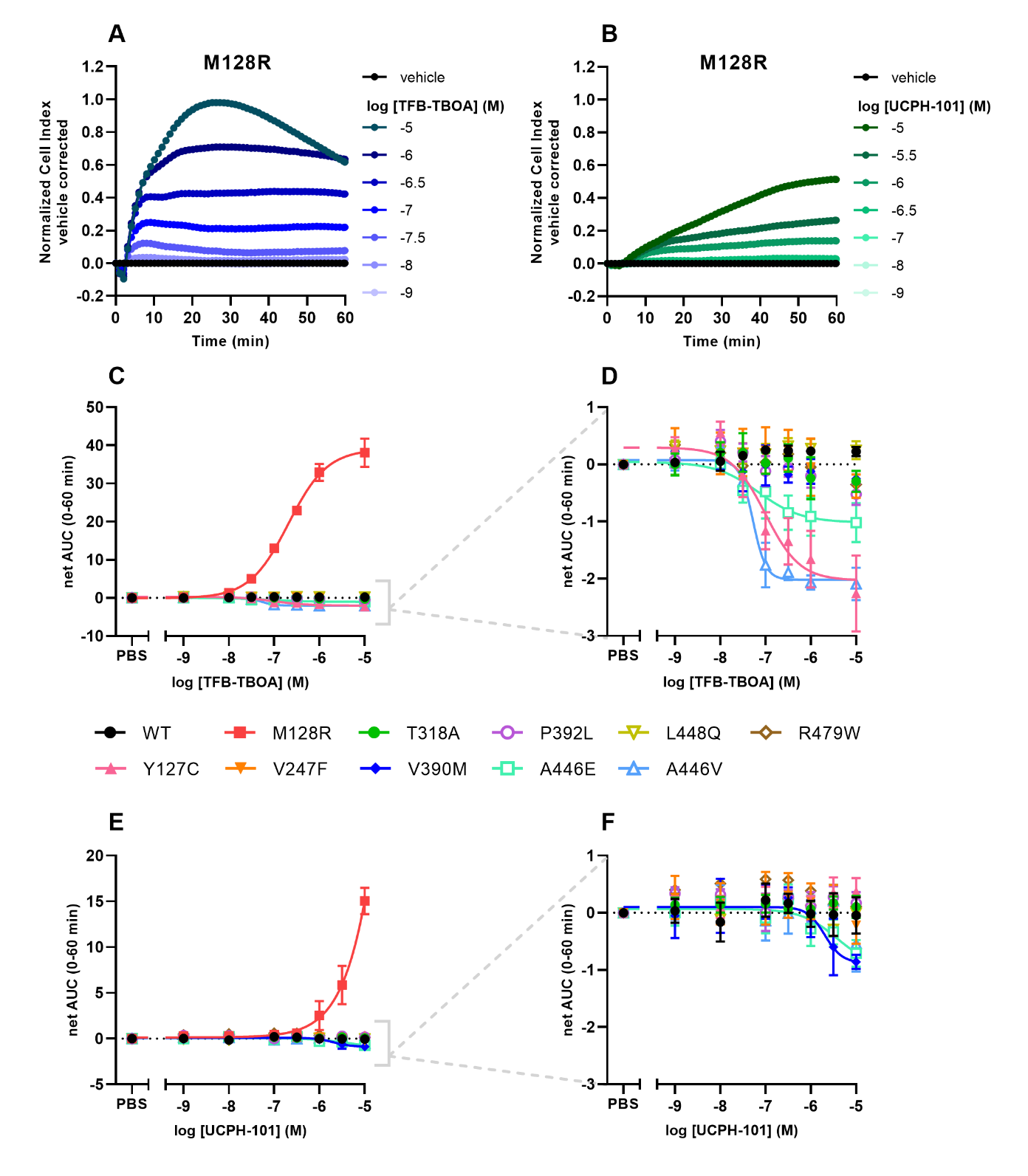
**

**Supplementary Figure 5.** Cellular responses of TFB-TBOA and UCPH-101 during pretreatment in an impedance-based phenotypic assay on EAAT_WT_ and mutant cells. (**A,B**) Vehicle-corrected normalized Cell Index traces of M128R cells pretreated with (**A**) TFB-TBOA or (**B**) UCPH-101 from a representative experiment. (**C**) Concentration-response curves of TFB-TBOA on M128R cells and (**D**) zoom-in on EAAT1_WT_ and other mutant cells. (**E**) Concentration-response curves of UCPH-101 on M128R cells and (**F**) zoom-in on EAAT1_WT_ and other mutant cells. Cellular response is expressed as the net AUC of the first 60 min after inhibitor pretreatment. Data are shown as the mean ± SEM of three individual experiments each performed in duplicate.

**
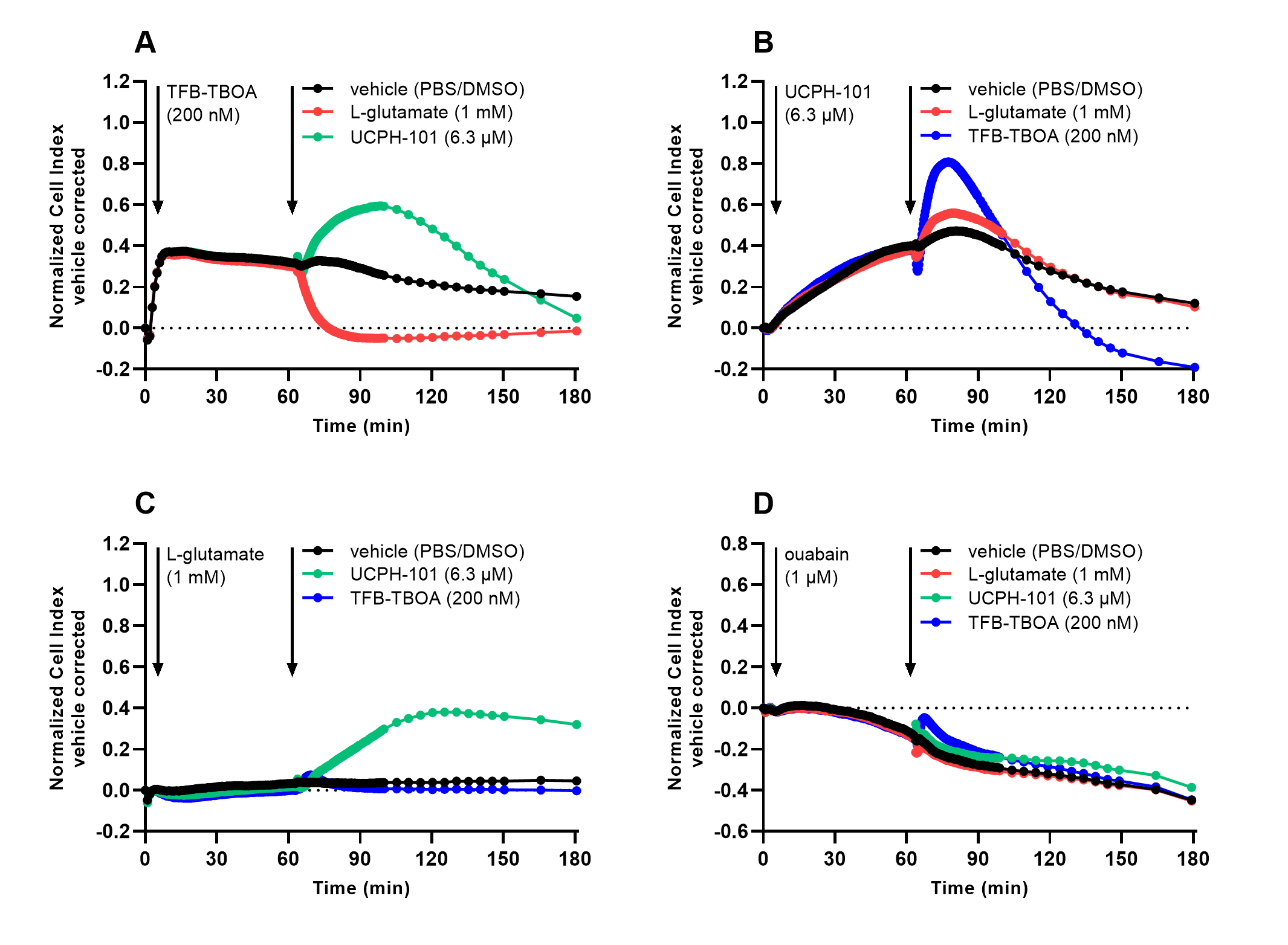
**

**Supplementary Figure 6.** Modulation of cellular responses by L-glutamate and inhibitors in an impedance-based phenotypic assay on M128R cells. (**A**) Pretreatment with EC_50_ (200 nM) TFB-TBOA and stimulation with vehicle, 1 mM L-glutamate or EC_50_ (6.3 µM) UCPH-101. (**B**) Pretreatment with 6.3 µM UCPH-101 and stimulation with vehicle, 1 mM L-glutamate or 200 nM TFB-TBOA. (**C**) Pretreatment with 1 mM L-glutamate and stimulation with vehicle, 6.3 µM UCPH-101 or 200 nM TFB-TBOA. (**D**) Pretreatment with 1 µM ouabain (Na^+^/K^+^-ATPase inhibitor) and stimulation with vehicle, 1 mM L-glutamate, 6.3 µM UCPH-101 or 200 nM TFB-TBOA. Data show vehicle-corrected normalized Cell Index traces of M128R cells pretreated for 60 min and subsequently stimulated for 120 min. Traces were normalized at the time point prior to pretreatment. Cells pretreated and stimulated with vehicle (PBS/DMSO) were used for vehicle-correction.


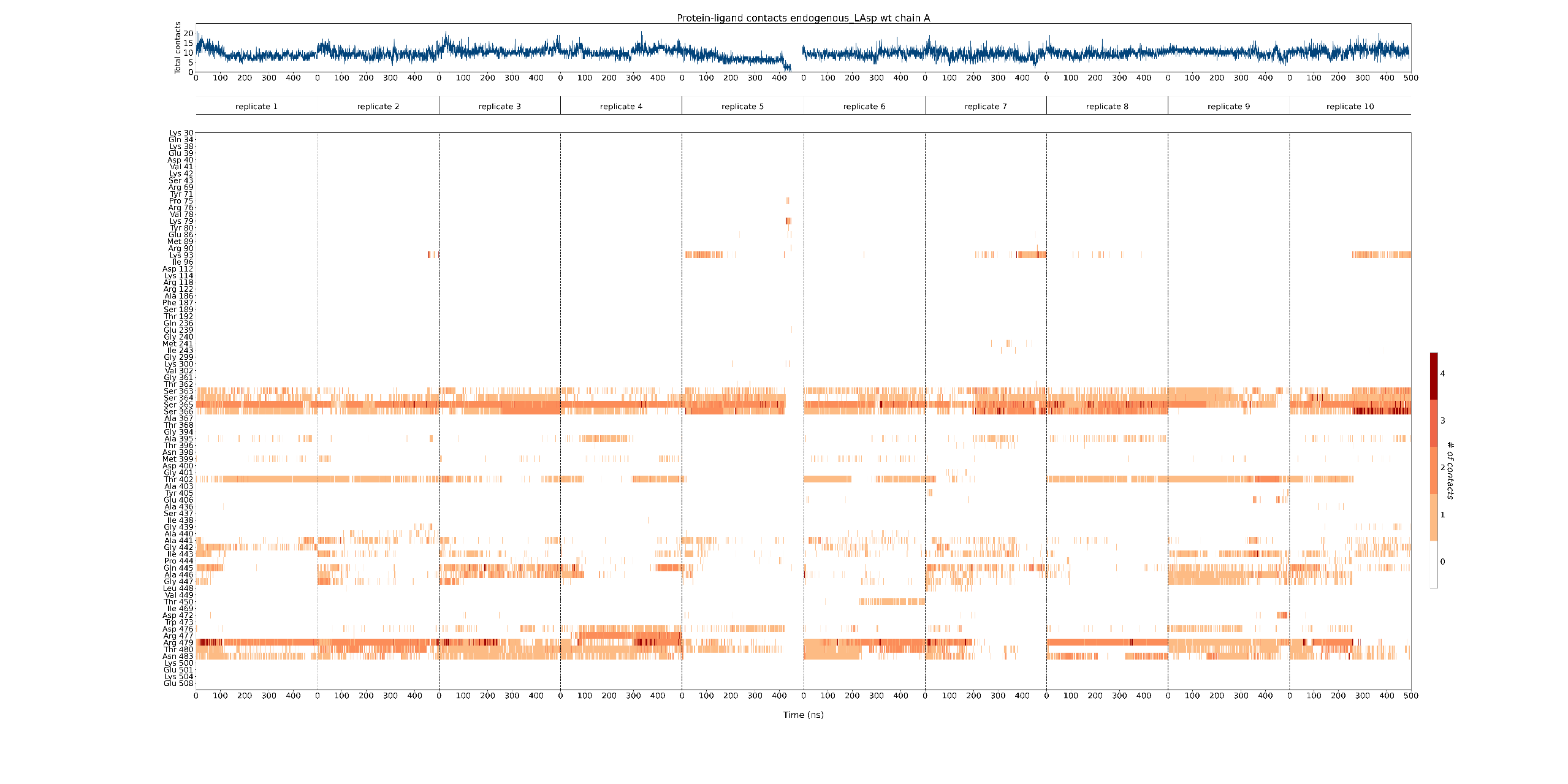


**Supplementary Figure 7.** Substrate – protein interactions across MD simulation replicates in EAAT1_WT_ chain A over time. In blue, the total number of L-Asp contacts with EAAT1 measured over the simulation time per replicate (500 ns). In different shades of orange, the number of contacts recorded with each residue at each simulation point.


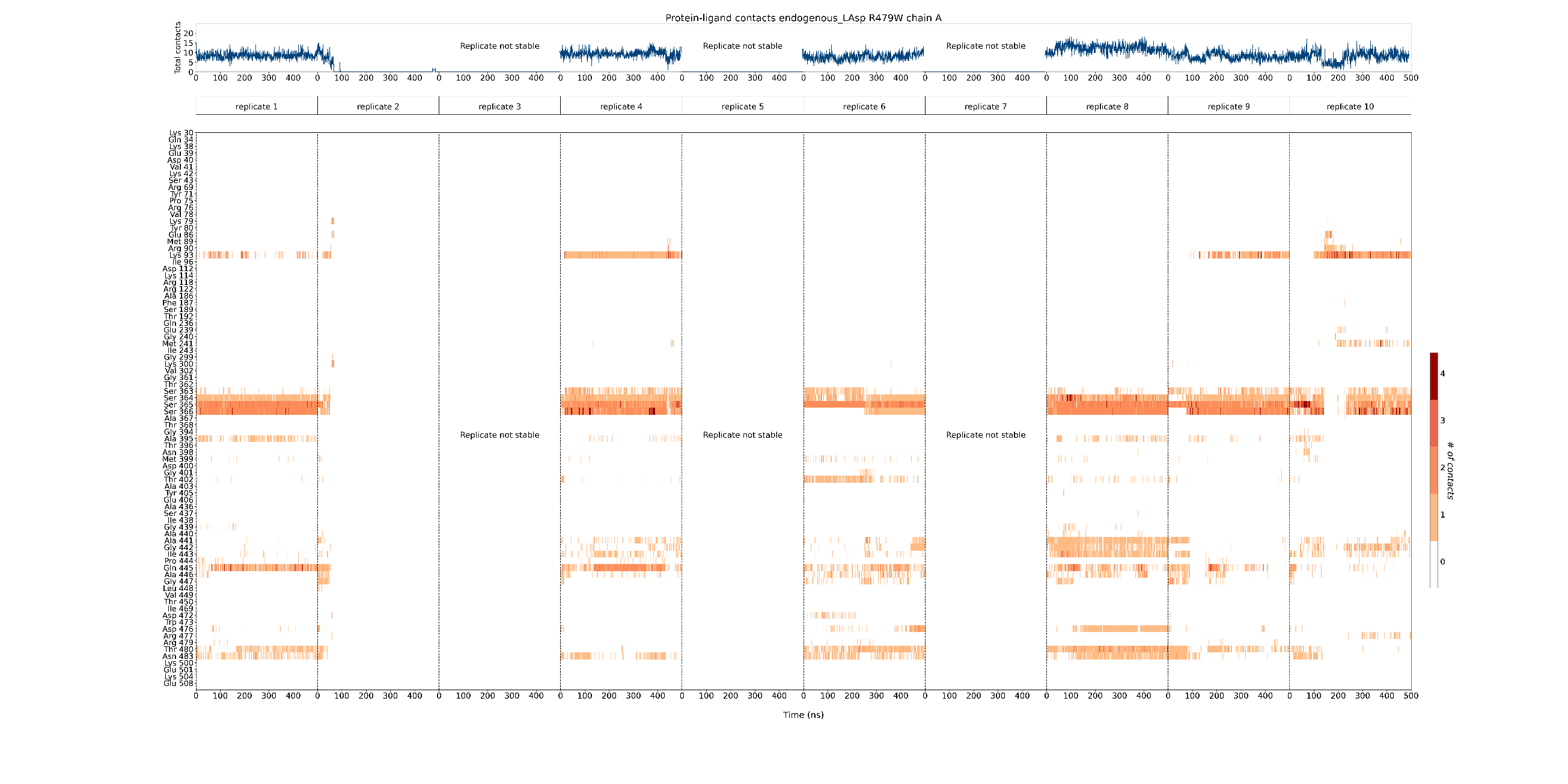


**Supplementary Figure 8.** Substrate – protein interactions across MD simulation replicates in EAAT mutant R479W chain A over time. In blue, the total number of L-Asp contacts with EAAT1 measured over the simulation time per replicate (500 ns). In different shades of orange, the number of contacts recorded with each residue at each simulation point. Replicates were protein RMSD reached 10 Å are labelled as “Replicate not stable” and the contacts are not reported.


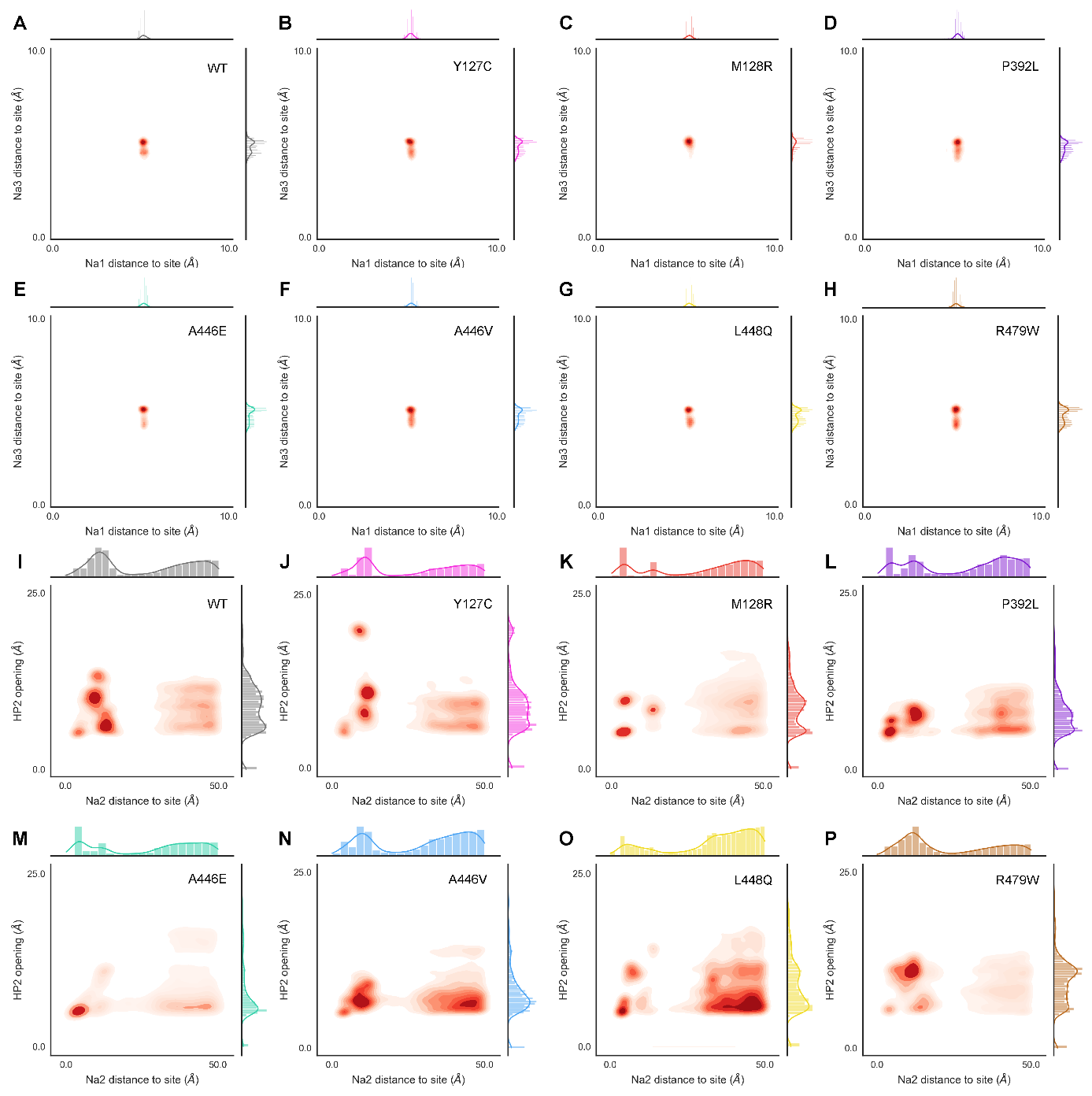


**Supplementary Figure 9.** Sodium ion coordination stability and HP2 domain opening sampling density derived from Molecular Dynamics simulations on EAAT1_WT_ and mutants. Sodium ion coordination stability is represented by the distance from the Na^+^ atom to the C α of one of its coordinating residues in sites Na1 (D487), Na2 (T396), and Na3 (D400). HP2 opening was calculated as the distance between S366 Cα (HP1 tip) and G442 Cα (HP2 tip). Sampling density was calculated across all frames in all replicates simulated for Na1-Na3 ion coordination stability **(A-H)** and Na2 coordination stability-HP2 opening **(I-P)**. Density was analyzed for both pairs in combination (inside the axes box) and independently (outside the axes) for EAAT1_wt_ **(A,I)** and mutants **(B-H;J-P)**.


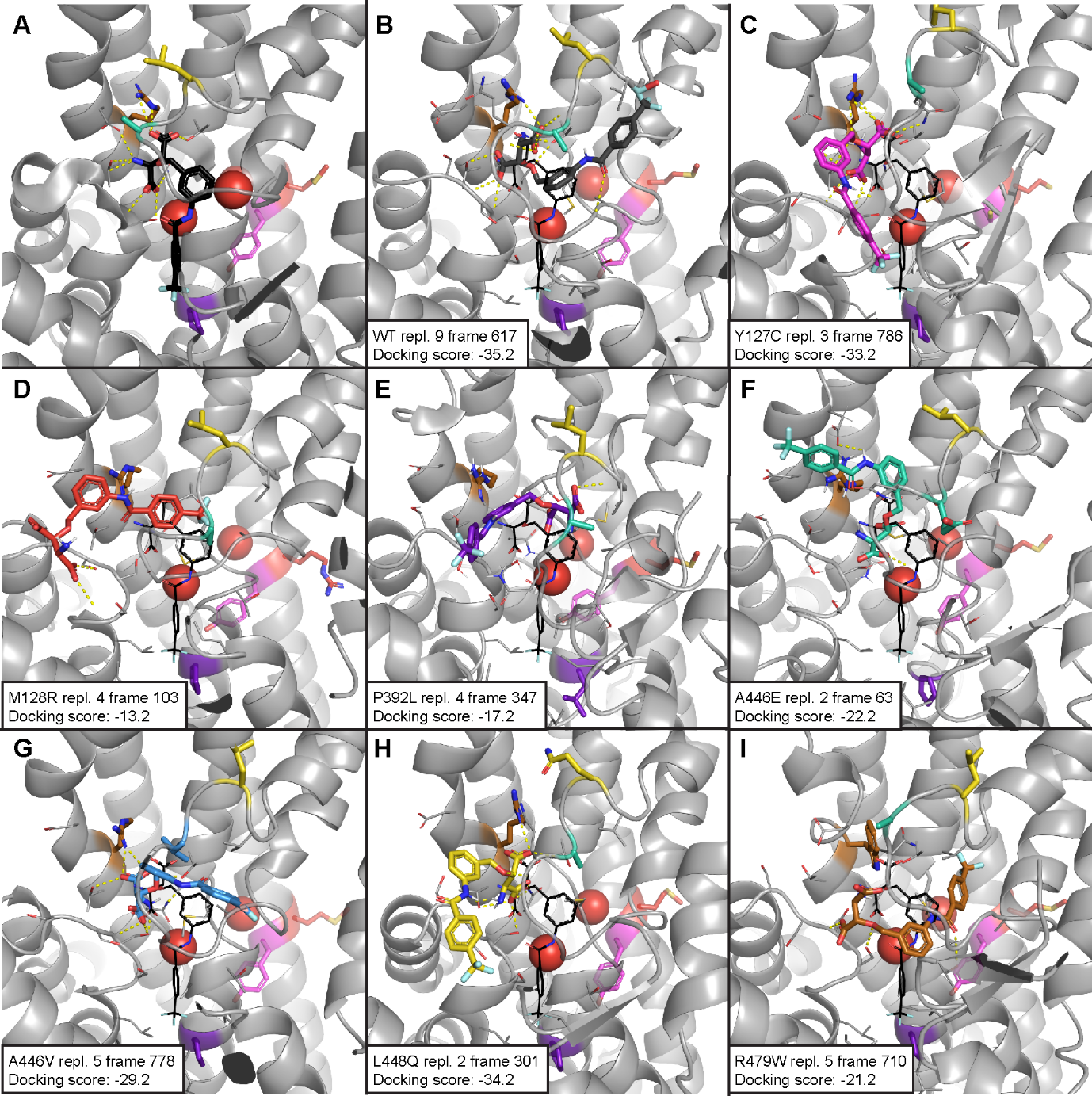


**Supplementary Figure 10.** Molecular docking top poses of orthosteric inhibitor TFB-TBOA in EAAT1 MD frames with most representative HP2 opening distances. Docking performed in chain A of a random selection of frames with the top five most common HP2 opening distances across all replicates and frames. TFB-TBOA binding pocket was derived from its co-crystalized pose in PDB 5MJU **(A)**, represented in black for reference next to the docking poses generated in EAAT1_WT_ **(B)** and mutants **(C-I)**. (Mutated) residues of interested are represented in the following colors: Y127 pink, M128 red, P392 purple, A446 green (or blue for A446V mutant), L448 yellow, and R479 brown. Coordinated Na^+^ ions are represented as red spheres. Hydrogen bonds are represented as dashed yellow lines.


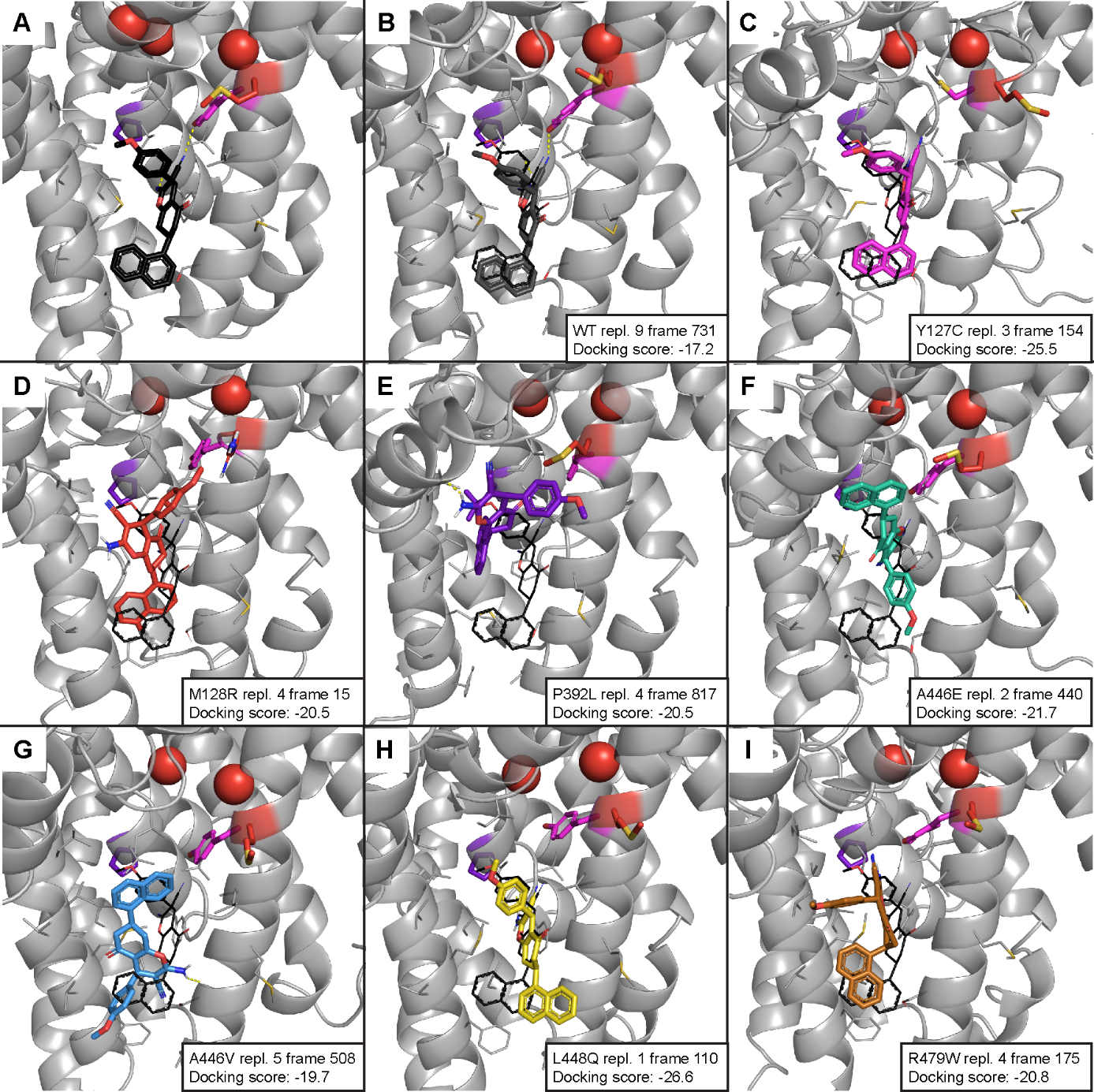


**Supplementary Figure 11.** Molecular docking top poses of allosteric inhibitor UCPH-101 in EAAT1 MD frames with most representative HP2 opening distances. Docking performed in chain A of a random selection of frames with the top five most common HP2 opening distances across all replicates and frames. UCPH-101 binding pocket was derived from its co-crystalized pose in PDB 7AWM **(A)**, represented in black for reference next to the docking poses generated in EAAT1_WT_ **(B)** and mutants **(C-I)**. (Mutated) residues of interested are represented in the following colors: Y127 pink, M128 red, P392 purple, A446 green (or blue for A446V mutant), L448 yellow, and R479 brown. Coordinated Na^+^ ions are represented as red spheres. Hydrogen bonds are represented as dashed yellow lines.


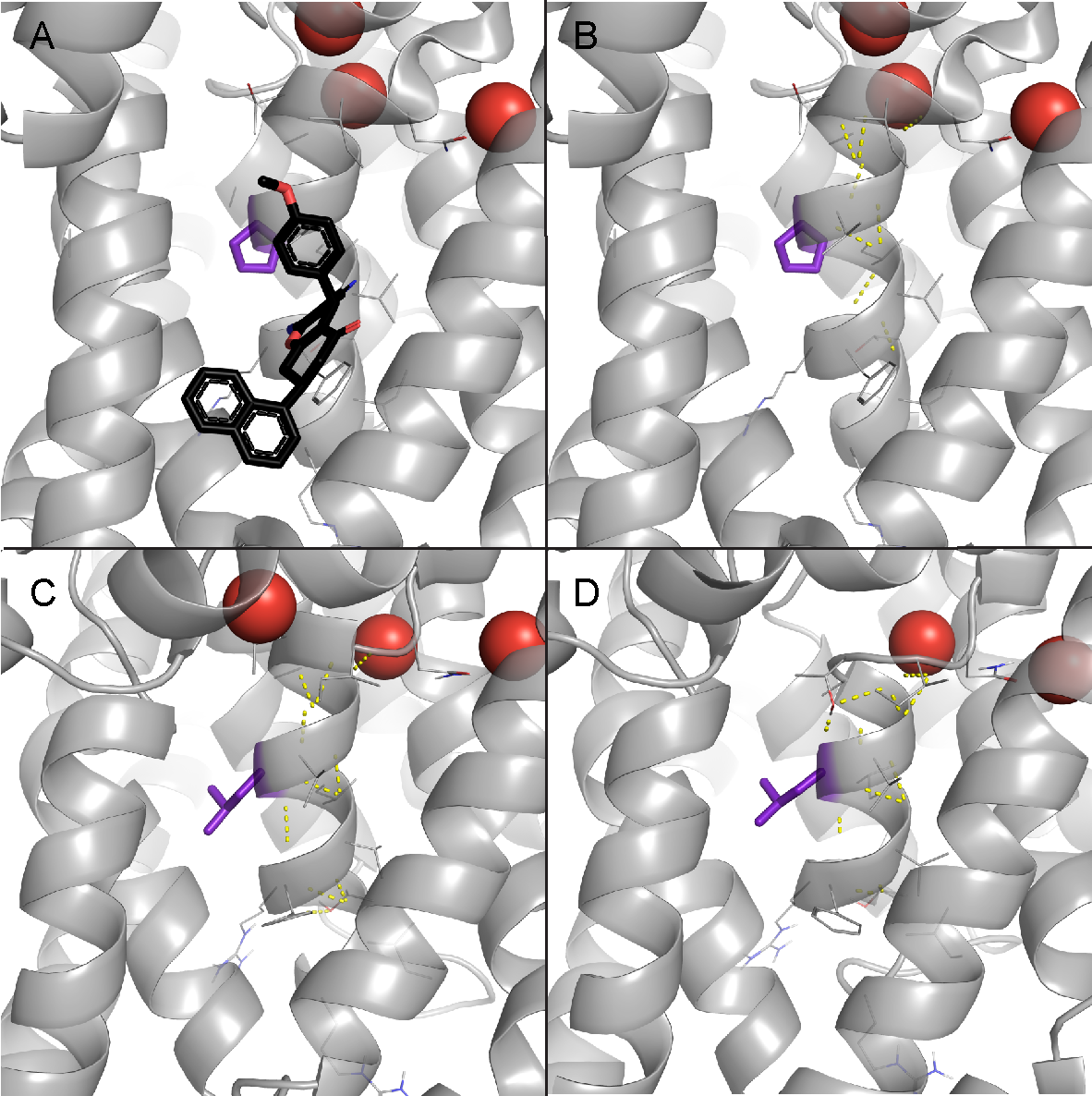


**Supplementary Figure 12.** Effect of P392L mutant in Pro-induced TM7a helix kink. **(A)** TM7a helix stabilizes the allosteric pocket where UCPH-101 inhibitor binds. Visualization in chain A of PDB 7AWM. **(B)** P392 (purple) induces a kink in the TM7a helix that is represented by a lack of an additional hydrogen bond (dashed yellow lines) in that helix turn. **(C-D)** P392L mutation reverts the Pro-induced kink, as represented by an additional hydrogen bond in that helix turn. Visualization in chain A of replicate 4 MD trajectory frames 347 **(C)** and 817 **(D)**.

**Supplementary Table 1.** Random seeds used to generate initial velocities in Molecular Dynamics simulations.

| **Mutant** | **Replicate 1** | **Replicate 2** | **Replicate 3** | **Replicate 4** | **Replicate 5** | **Replicate 6** | **Replicate 7** | **Replicate 8** | **Replicate 9** | **Replicate 10** |
| --- | --- | --- | --- | --- | --- | --- | --- | --- | --- | --- |
| **WT** | 1613 | 1825 | 8414 | 8636 | 6037 | 8843 | 5519 | 5522 | 973 | 9694 |
| **Y127C** | 5409 | 5655 | 9816 | 9194 | 2819 | 5369 | 3230 | 5695 | 2910 | 6457 |
| **M128R** | 112 | 8172 | 8063 | 4417 | 9724 | 4479 | 4263 | 6945 | 668 | 2947 |
| **P392L** | 8167 | 3981 | 2959 | 52 | 5364 | 7026 | 1533 | 5596 | 3822 | 6974 |
| **A446E** | 782 | 9717 | 8228 | 1096 | 3085 | 2107 | 3786 | 8496 | 1711 | 5788 |
| **A446V** | 7735 | 3445 | 1885 | 3556 | 6824 | 9192 | 4487 | 2489 | 4094 | 9957 |
| **L448Q** | 7248 | 2175 | 8437 | 8704 | 3512 | 2870 | 4162 | 2289 | 78 | 3219 |
| **R479W** | 8385 | 4603 | 128 | 9635 | 5711 | 1994 | 1530 | 7953 | 3132 | 4046 |

**Supplementary Table 2.** Frames with the five most common HP2 opening distances across replicates of EAAT1 chain A MD simulations selected for docking.

| **Mutant** | **Frame 1** | **Frame 2** | **Frame 3** | **Frame 4** | **Frame 5** |
| --- | --- | --- | --- | --- | --- |
| **WT** | Replicate 1 frame 203 | Replicate 9 frame 125 | Replicate 9 frame 231 | Replicate 9 frame 617 | Replicate 9 frame 731 |
| **Y127C** | Replicate 1 frame 522 | Replicate 3 frame 154 | Replicate 3 frame 618 | Replicate 3 frame 786 | Replicate 3 frame 892 |
| **M128R** | Replicate 4 frame 15 | Replicate 4 frame 103 | Replicate 4 frame 546 | Replicate 4 frame 769 | Replicate 4 frame 941 |
| **P392L** | Replicate 4 frame 347 | Replicate 4 frame 609 | Replicate 4 frame 817 | Replicate 4 frame 880 | Replicate 4 frame 971 |
| **A446E** | Replicate 2 frame 6 | Replicate 2 frame 440 | Replicate 8 frame 63 | Replicate 8 frame 386 | Replicate 10 frame 254 |
| **A446V** | Replicate 1 frame 163 | Replicate 5 frame 35 | Replicate 5 frame 508 | Replicate 5 frame 530 | Replicate 5 frame 778 |
| **L448Q** | Replicate 1 frame 110 | Replicate 2 frame 301 | Replicate 5 frame 82 | Replicate 5 frame 455 | Replicate 6 frame 93 |
| **R479W** | Replicate 4 frame 175 | Replicate 5 frame 431 | Replicate 5 frame 491 | Replicate 5 frame 550 | Replicate 5 frame 710 |

**Supplementary Table 3.** Orthosteric pocket properties across the five random frames selected per mutant for 4D docking. Pockets were predicted and characterized using the ICM pocket finder tool and visually inspected to match the orthosteric pocket (*: some orthosteric pockets seem to be divided into two pockets in ICM). Pocket properties are volume (Å^3^), Hydrophobicity (representing the percentage of the pocket surface in contact with hydrophobic residues, ranges from 0 to 1), Buriedness (calculated based on solvent accessibility, ranges from 0.5 – completely open and surface flat – to 1.0 – completely buried), and DLID (Merck's Drug-like density score (see Sheridan et al JCIM 2010). Values above zero and those with slightly negative values are considered "druggable").

| **Frame** | **Pocket** | **Volume** | **Hydrophobicity** | **Buriedness** | **DLID** |
| --- | --- | --- | --- | --- | --- |
| 7AWM | Orthosteric | 147.01 | 0.59 | 1.00 | 0.26 |
| 5MJU | Orthosteric | 607.78 | 0.42 | 0.69 | -0.31 |
| wt_1A_203 | Orthosteric | 467.11 | 0.62 | 0.90 | 0.78 |
| wt_9A_125 | Orthosteric | 462.99 | 0.63 | 0.90 | 0.79 |
| wt_9A_231 | Orthosteric | 400.09 | 0.58 | 0.86 | 0.44 |
| wt_9A_617 | Orthosteric | 493.75 | 0.59 | 0.84 | 0.52 |
| wt_9A_731 | Orthosteric | 722.43 | 0.65 | 0.88 | 1.11 |
| Y127C_1A_522 | Orthosteric | 784.45 | 0.51 | 0.76 | 0.37 |
| Y127C_3A_154 | Orthosteric | 475.74 | 0.48 | 0.81 | 0.15 |
| Y127C_3A_618 | Orthosteric | 596.56 | 0.62 | 0.94 | 1.13 |
| Y127C_3A_786 | Orthosteric | 389.43 | 0.55 | 0.90 | 0.48 |
| Y127C_3A_892 | Orthosteric | 437.20 | 0.44 | 0.80 | -0.06 |
| M128R_4A_15 | Orthosteric | 187.94 | 0.81 | 0.99 | 0.92 |
| M128R_4A_103 | Orthosteric | 388.81 | 0.64 | 0.87 | 0.58 |
| M128R_4A_546 | Orthosteric | 504.84 | 0.60 | 0.89 | 0.77 |
| M128R_4A_769 | Orthosteric | 194.63 | 0.73 | 0.97 | 0.66 |
| M128R_4A_769 | Orthosteric* | 101.61 | 0.32 | 0.63 | -2.06 |
| M128R_4A_941 | Orthosteric | 186.41 | 0.81 | 0.99 | 0.91 |
| P392L_4A_347 | Orthosteric | 341.24 | 0.48 | 0.76 | -0.29 |
| P392L_4A_609 | Orthosteric | 395.64 | 0.61 | 0.89 | 0.59 |
| P392L_4A_817 | Orthosteric | 568.70 | 0.61 | 0.90 | 0.91 |
| P392L_4A_880 | Orthosteric | 505.91 | 0.57 | 0.84 | 0.50 |
| P392L_4A_971 | Orthosteric | 341.05 | 0.47 | 0.76 | -0.32 |
| A446E_2A_63 | Orthosteric | 670.60 | 0.55 | 0.78 | 0.45 |
| A446E_2A_63 | Orthosteric* | 135.49 | 0.72 | 0.96 | 0.33 |
| A446E_2A_440 | Orthosteric | 740.40 | 0.61 | 0.90 | 1.12 |
| A446E_8A_69 | Orthosteric | 360.90 | 0.53 | 0.89 | 0.34 |
| A446E_8A_386 | Orthosteric | 584.50 | 0.52 | 0.84 | 0.49 |
| A446E_10A_254 | Orthosteric | 670.88 | 0.55 | 0.78 | 0.43 |
| A446E_10A_254 | Orthosteric* | 135.76 | 0.72 | 0.96 | 0.34 |
| A446V_1A_163 | Orthosteric | 405.01 | 0.58 | 0.87 | 0.48 |
| A446V_5A_35 | Orthosteric | 613.30 | 0.59 | 0.81 | 0.57 |
| A446V_5A_508 | Orthosteric | 323.34 | 0.48 | 0.79 | -0.24 |
| A446V_5A_530 | Orthosteric | 304.85 | 0.59 | 0.84 | 0.17 |
| A446V_5A_778 | Orthosteric | 631.34 | 0.56 | 0.81 | 0.52 |
| L448Q_1A_110 | Orthosteric | 482.15 | 0.54 | 0.87 | 0.51 |
| L448Q_2A_301 | Orthosteric | 327.78 | 0.61 | 0.94 | 0.66 |
| L448Q_5A_82 | Orthosteric | 557.76 | 0.64 | 0.91 | 1.03 |
| L448Q_5A_455 | Orthosteric | 373.75 | 0.61 | 0.85 | 0.40 |
| L448Q_6A_93 | Orthosteric | 553.21 | 0.66 | 0.91 | 1.05 |
| R479W_4A_175 | Orthosteric* | 571.68 | 0.62 | 0.82 | 0.65 |
| R479W_4A_175 | Orthosteric | 234.19 | 0.56 | 0.86 | 0.02 |
| R479W_5A_431 | Orthosteric | 529.61 | 0.62 | 0.86 | 0.71 |
| R479W_5A_491 | Orthosteric | 714.29 | 0.57 | 0.85 | 0.80 |
| R479W_5A_550 | Orthosteric | 565.14 | 0.53 | 0.78 | 0.24 |
| R479W_5A_710 | Orthosteric | 578.60 | 0.61 | 0.82 | 0.63 |
| R479W_5A_710 | Orthosteric* | 235.80 | 0.54 | 0.84 | -0.12 |

**Supplementary Table 4.** Allosteric (UCPH-101) pocket properties across the five random frames selected per mutant for 4D docking. Pockets were predicted and characterized using the ICM pocket finder tool and visually inspected to match the allosteric pocket. The allosteric pocket was missing in some frames, which are not recorded in the table. Pocket properties are volume (Å^3^), Hydrophobicity (representing the percentage of the pocket surface in contact with hydrophobic residues, ranges from 0 to 1), Buriedness (calculated based on solvent accessibility, ranges from 0.5 – completely open and surface flat – to 1.0 – completely buried), and DLID (Merck's Drug-like density score (see Sheridan et al JCIM 2010). Values above zero and those with slightly negative values are considered "druggable").

| **Frame** | **Pocket** | **Volume** | **Hydrophobicity** | **Buriedness** | **DLID** |
| --- | --- | --- | --- | --- | --- |
| 5MJU | Allosteric | 129.07 | 0.67 | 0.77 | -0.55 |
| 7AWM | Allosteric | 114.40 | 0.41 | 0.55 | -2.10 |
| wt_9A_231 | Allosteric | 119.49 | 0.63 | 0.65 | -1.17 |
| wt_9A_617 | Allosteric | 166.22 | 0.66 | 0.72 | -0.58 |
| wt_9A_731 | Allosteric | 110.85 | 0.40 | 0.53 | -2.25 |
| Y127C_3A_154 | Allosteric | 165.31 | 0.62 | 0.68 | -0.84 |
| Y127C_3A_618 | Allosteric | 235.42 | 0.72 | 0.73 | -0.13 |
| Y127C_3A_786 | Allosteric | 169.85 | 0.73 | 0.80 | -0.08 |
| Y127C_3A_892 | Allosteric | 166.86 | 0.64 | 0.69 | -0.77 |
| M128R_4A_769 | Allosteric | 108.86 | 0.51 | 0.57 | -1.85 |
| P392L_4A_347 | Allosteric | 287.89 | 0.71 | 0.78 | 0.18 |
| P392L_4A_817 | Allosteric | 160.23 | 0.54 | 0.61 | -1.31 |
| P392L_4A_880 | Allosteric | 177.89 | 0.67 | 0.72 | -0.50 |
| P392L_4A_971 | Allosteric | 290.36 | 0.72 | 0.78 | 0.20 |
| A446E_2A_440 | Allosteric | 119.92 | 0.49 | 0.59 | -1.74 |
| A446E_8A_69 | Allosteric | 118.66 | 0.37 | 0.46 | -2.54 |
| R479W_5A_550 | Allosteric | 106.79 | 0.50 | 0.55 | -1.92 |
